# Role of mutations and post-translational modifications in systemic AL amyloidosis studied by cryo-EM

**DOI:** 10.1038/s41467-021-26553-9

**Published:** 2021-11-05

**Authors:** Lynn Radamaker, Sara Karimi-Farsijani, Giada Andreotti, Julian Baur, Matthias Neumann, Sarah Schreiner, Natalie Berghaus, Raoul Motika, Christian Haupt, Paul Walther, Volker Schmidt, Stefanie Huhn, Ute Hegenbart, Stefan O. Schönland, Sebastian Wiese, Clarissa Read, Matthias Schmidt, Marcus Fändrich

**Affiliations:** 1 Institute of Protein Biochemistry, Ulm University, 89081 Ulm, Germany; 2 Institute of Stochastics, Ulm University, 89081 Ulm, Germany; 3 Medical Department V, Section of Multiple Myeloma, Heidelberg University Hospital, 69120 Heidelberg, Germany; 4 Department of Asia-Africa-Studies, Middle Eastern History and Culture, University of Hamburg, 20148 Hamburg, Germany; 5 Central Facility for Electron Microscopy, Ulm University, 89081 Ulm, Germany; 6 Medical Department V, Amyloidosis Center, Heidelberg University Hospital, 69120 Heidelberg, Germany; 7 Core Unit Mass Spectrometry and Proteomics, Medical Faculty, Ulm University, 89081 Ulm, Germany; 8 Institute of Virology, Ulm University Medical Center, 89081 Ulm, Germany

**Keywords:** Structural biology, Protein aggregation, Cryoelectron microscopy, Cryoelectron tomography

## Abstract

Systemic AL amyloidosis is a rare disease that is caused by the misfolding of immunoglobulin light chains (LCs). Potential drivers of amyloid formation in this disease are post-translational modifications (PTMs) and the mutational changes that are inserted into the LCs by somatic hypermutation. Here we present the cryo electron microscopy (cryo-EM) structure of an ex vivo λ1-AL amyloid fibril whose deposits disrupt the ordered cardiomyocyte structure in the heart. The fibril protein contains six mutational changes compared to the germ line and three PTMs (disulfide bond, N-glycosylation and pyroglutamylation). Our data imply that the disulfide bond, glycosylation and mutational changes contribute to determining the fibril protein fold and help to generate a fibril morphology that is able to withstand proteolytic degradation inside the body.

## Abstract (web summary)

Systemic AL amyloidosis is caused by misfolding of immunoglobulin light chains (LCs) but how post-translational modifications (PTMs) of LCs influence amyloid formation is not well understood. Here, the authors present the cryo-EM structure of an AL amyloid fibril derived from the heart tissue of a patient that is partially pyroglutamylated, N-glycosylated and contains an intramolecular disulfide bond. Based on their structure and biochemical experiments the authors conclude that the mutational changes, disulfide bond and glycosylation determine the fibril protein fold and that glycosylation protects the fibril core from proteolytic degradation.

## Introduction

Systemic AL amyloidosis is defined by the formation of amyloid fibrils by immunoglobulin light chains (LCs)^1,2^. These fibrils can deposit at multiple sites in the body where they can lead to severe impairment of vital organ functions. Untreated patients with a prominent heart involvement show a high risk of death with a median survival of only seven months after their initial diagnosis^3,4^. Due to their natural function as part of B-cell receptors and antibodies, LCs are hypervariable proteins. This variability arises from the genetic recombination of variable (V), joining (J), and constant (C) gene segments that are encoded in the germ line (GL), somatic hypermutation, and the junctional diversity at the V/J interface in the course of V/J recombination^5^. Altogether, there are 63–71 functional V (34–38 V_κ_ and 29–33 V_λ_), 9–10 J (5 J_κ_ and 4–5 J_λ_), and 5–6 C (1 C_κ_ and 4–5 C_λ_) segments encoded within the GL^6^ of which *IGLV6-57*, *IGLV3-01*, *IGLV2-14*, and *IGKV1-33* were found to be overrepresented in systemic AL amyloidosis^7–10^. These findings and the fact that usage of *IGLV1-44* is associated with cardiac involvement^11^ imply that the LC primary structure is a key determinant for the development of amyloidosis.

Further support for this view comes from observations that the mutational changes that are inserted into the LCs during somatic hypermutation affect the kinetics of amyloid fibril formation in vitro^1^. The effect of these mutations has frequently been attributed to alterations in the biophysical properties of the natively folded, globular LCs, such as to a decreased thermodynamic stability^12,13^, to increased conformational dynamics^14–16^, or to local structural changes in a key region of the protein^14–16^. In other cases, it was suggested that the mutations may affect the formation of specific folding or misfolding intermediates^14,17^ or the stability or structure of the resulting amyloid fibril^18,19^. Post-translational modifications (PTMs) may further modulate the effects of the inserted mutations. Several studies have shown that AL fibril proteins can contain PTMs like disulfide bonds^20–23^, N-terminal pyroglutamate (Pyro-Glu) modifications^24,25^, or glycosylations^26,27^. Glycosylation was found to be overrepresented in AL patients, indicating that this PTM contributes to the pathogenesis in AL amyloidosis^26,28^. Yet, the mechanism by which glycosylation affects fibril formation or AL pathogenesis has so far remained unclear.

To shed light on the structural effects of PTMs and mutational changes on the fibril state, we have determined the structure of an AL amyloid fibril with cryoelectron microscopy (cryo-EM), which is partially pyroglutamylated, N-glycosylated and modified by an intramolecular disulfide bond. The observed fibril structure is different from previously described, nonglycosylated AL amyloid fibrils^21–23^, consistent with the patient-specific nature of this disease. The mutational changes are clustered into two topological regions of natively folded variable LC domains, but dispersed throughout the fold of the fibril protein with no obvious structural preference.

## Results

### FOR001 amyloid fibrils are structurally rigid and disrupt the ordered architecture of the heart muscle

**Fig. 1:**
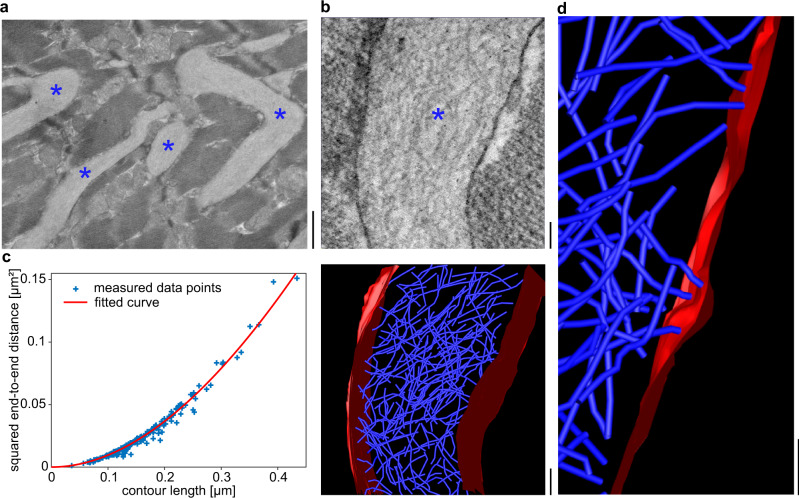
Tissue deposits of FOR001 amyloid fibrils. **a** SEM overview of FOR001 heart tissue. The fibril deposits between cardiomyocytes are marked with blue asterisks. Scale bar: 1 μm. **b** STEM tomogram. Top: virtual section. The fibril deposit is marked with a blue asterisk. Bottom: rendered tomogram of the fibril deposit. Blue: amyloid fibrils. Red: membranes. Scale bar: 100 nm. **c** Analysis of the persistence length based on a fit of the plot showing the squared end-to-end distance versus the contour length using Eq. (1). The blue symbols show the measured data points (*n* = 195) and the red line the fit. **d** Region of the tomogram, showing the interactions of the fibrils (blue) with the cardiomyocyte membrane (red). Scale bar 100 nm.

The presently analyzed fibrils were extracted from the heart tissue of patient FOR001, who suffered from advanced cardiac AL amyloidosis and underwent a heart transplantation at the age of 51 years. Analysis of sections of cardiac tissue with scanning electron microscopy (SEM) demonstrates that the fibrils form large-sized, extracellular amyloid deposits that infiltrate and disrupt the ordered structure of the cardiomyocytes (Fig. 1a, b). Using scanning transmission electron microscopy (STEM), we obtained tomograms of the three-dimensional (3D) structure of the cardiac amyloid deposits (Fig. 1b). The deposits are composed of haphazardly arranged fibrils that show a width of ~9 nm. Quantification of the fibril end-to-end distances and contour lengths allowed us to determine the fibril persistence length at 0.74 ± 0.08 μm and its bending rigidity at 3.1 × 10^−27^ ± 3 × 10^−28^ Nm^2^ (Fig. 1c). The persistence length corresponds to values reported in the literature^29,30^ for amyloid fibrils, which vary between 16 nm and 18.5 μm, indicating that FOR001 amyloid fibrils and their deposits are structurally rigid. The fibrils in these deposits interact with the surfaces of adjacent cardiomyocytes, and these interactions occur mainly via the fibril tips (Fig. 1d), and only rarely via the fibril lateral surfaces. In some cases, we find deformations in the plasma membrane associated with the focal contact points of the fibrils (Fig. 1d). The fibrils impede the contractile function of the heart, suggesting that clearance of the amyloid may help to restore cardiac function. While there is evidence that the patient’s health condition is also defined by circulating amyloid precursors in the serum^31^, our observations underpin the view that the amyloid deposits are damaging to the patient.

### Cryo-EM structure of the FOR001 AL amyloid fibril

**Fig. 2:**
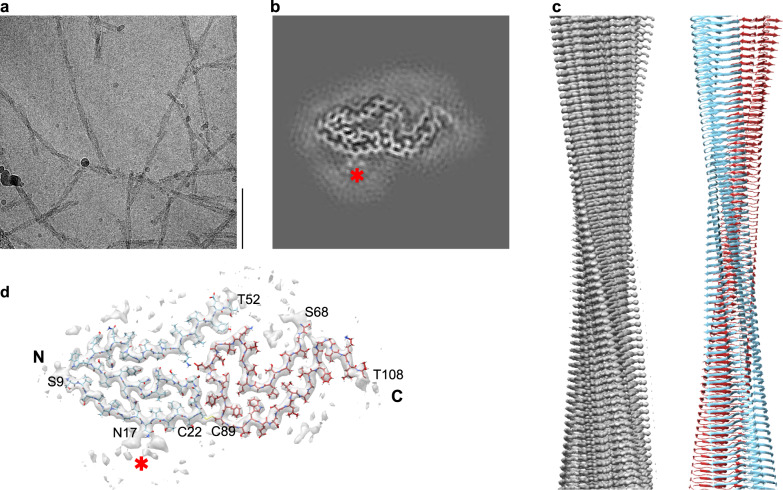
Cryo-EM structure of the FOR001 AL amyloid fibril. **a** Cryo-EM image of FOR001 amyloid fibrils. Scale bar is 100 nm. The dataset consists of 3033 micrographs. **b** Cross section of the map obtained by summing five central slices. **c** Side view of the map (left) and molecular model (right), showing the left-handed fibril twist. **d** Cross section of the map with the molecular model overlaid. The color coding of the model is the same in panels (**c**) and (**d**), that is, light blue refers to the N-terminal segment of the ordered fibril protein (residues Ser9–Thr52), while the deep-red segment refers to the C-terminal segment (residues Ser68–Thr108). The two segments are cross-linked through a disulfide between Cys22 and Cys89. The red star in (**b**) and (**d**) indicates the glycosylation site at Asn17.

To investigate the structure of the extracted FOR001 AL fibrils, we imaged them using cryo-EM (Fig. 2a). The images show that approximately 75% of the fibrils seen in the micrographs belong to one dominant morphology. This fibril morphology is defined by a width of ~9 nm, which agrees with the width measured by electron tomography (see above), and a crossover distance of ~55 nm, as measured from the recorded images. 3D reconstruction of the fibril images yielded a 3D map of the dominant morphology with a resolution of 3.1 Å (Supplementary Fig. 1a, Supplementary Table 1), while the remaining minor morphologies could not be reconstructed. The rise and twist of the reconstructed fibril are 4.76 Å and −1.46°, the pitch is 117 nm (Supplementary Table 1). The fibril consists of a single protofilament (C1 symmetry, Fig. 2b, c). The fitted molecular model (Fig. 2d) has a model resolution of 3.1 Å, (Supplementary Table 1). Projections of its density onto the y–z plane correspond well to the two-dimensional (2D) class averages (Supplementary Fig. 1b). The handedness of the fibrils in this sample was determined by platinum side-shadowing, which showed a left-handed twist (Supplementary Fig. 2). The ordered core of the fibril consists of two segments that extend from Ser9 to Thr52 and Ser68 to Thr108 (Fig. 2d). For residues Gln1–Pro8, Asp53–Lys67, and Val109–Ser118, which are present in the fibril as shown by mass spectrometry (MS) (see below), no well-defined density could be discerned in our map, suggesting that they are structurally disordered. The fibril protein contains an intramolecular disulfide that connects residues Cys22 and Cys89 (Fig. 2d).

### Chemical interactions stabilizing the fibril protein fold

**Fig. 3:**
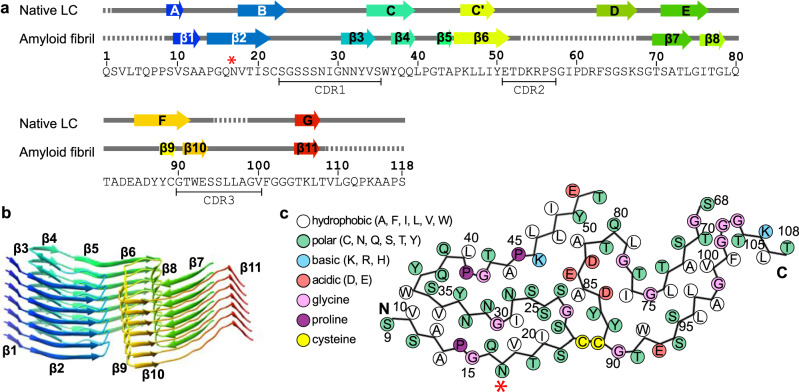
Location of the secondary structural elements and mutational sites in the fibril structure. **a** Amino acid sequence of the FOR001 fibril protein and secondary structural elements of the FOR001 fibril protein (PDB 7NSL) and of a crystal structure of a natively folded LC (PDB 4ODH 10.2210/pdb4ODH/pdb) containing an IGLV1-51*02 segment. Arrows indicate β-strands in the structure, rainbow-colored from N (blue) to C terminus (red). Dotted lines represent disordered segments. Red star: location of the glycosylation. **b** Stack of seven protein layers of the fibril, showing the β-strands β1–β11 with the same coloring as in (**a**). **c** Schematic representation of the fibril protein fold. Red star: location of the glycosylation.

Despite being dominated by β-sheet conformation, the fibril protein structure is profoundly different from the structure of a natively folded LC (Fig. 3a) and encompasses eleven parallel cross-β-sheets (β1–β11) that are formed by residues Val10–Ala12, Pro14–Ser21, Asn31–Val34, Tyr37–Gln39, Thr43–Ala44, Pro45–Glu51, Thr70–Leu74, Ile76–Gly78, Tyr88–Cys89, Thr91–Glu93, and Thr105–Leu107 (Fig. 3a, b). The β-strands in these sheets interact in the direction of the fibril z axis through backbone hydrogen bonds as well as through side-chain interactions, including the stacking of aromatic and polar residues (Supplementary Fig. 3a, b). The fibril protein fold is defined by buried electrostatic interactions, such as between Glu84 and Lys46 (Supplementary Fig. 3c), and by several buried patches of hydrophobic residues, such as the one formed by residues Val10, Ala12, Val34, and Trp36 (Fig. 3c). The fibril quaternary structure is stabilized by an interlocking of the protomers in the direction of the z-axis that is caused by a 14-Å height change of the fibril protein backbone (Supplementary Fig. 3d).

### Primary structure of the FOR001 fibril protein

The FOR001 fibril protein sequence was determined with electrospray-ionization MS (Supplementary Fig. 4). DNA sequencing of bone-marrow-derived cDNA was also attempted but failed to produce a sequence that matched the sequence obtained by direct sequencing of the fibril protein. Three major fibril protein species were revealed by MS (Supplementary Fig. 5a). Two of these could be assigned to the LC fragments Pyro–Glu1 to Ser118 and Ser2 to Ser118 (Supplementary Fig. 5b), both containing a disulfide bond. The third species corresponds to the fragment containing residues Val3–Ser118 and a disulfide bond, although alternative mass assignments are also possible for the recorded MS peak (Supplementary Fig. 5b). These analyses reveal two PTMs: a disulfide bond and a pyroglutamylation (Supplementary Fig. 5c) that affects only a fraction of the fibril proteins. The third PTM of the fibril protein is an ~2-kDa N-glycosylation, which is demonstrated by an electrophoretic band shift of the refolded FOR001 fibril protein upon addition of N-glycosidase but not with O-glycosidase (Supplementary Fig. 5d). The carbohydrate can be seen as extra density within our 3D map that could not be assigned to the polypeptide chain (Fig. 2b, d, red star). It protrudes from residue Asn17, which forms part of the only canonical N-glycosylation site (Asn–Xaa–Thr/Ser) in the fibril protein sequence.

### Identification of the GL segments and mutational changes

**Fig. 4:**
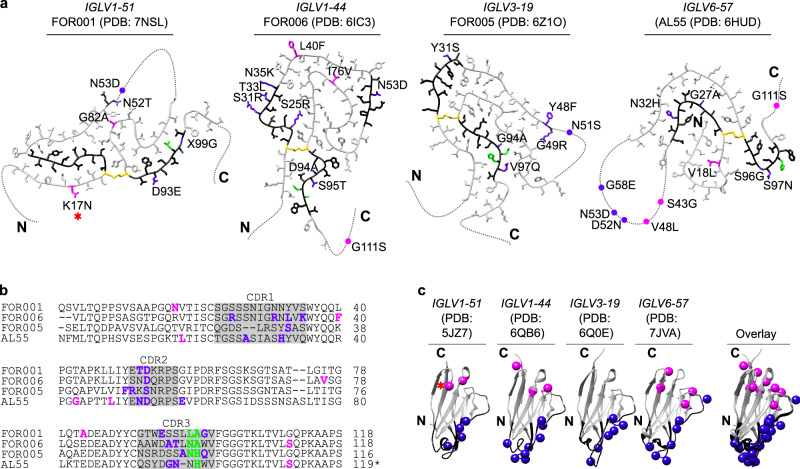
Location of the mutations in known AL amyloid fibrils and natively folded V_L_ domains. **a** Location of the mutations in known AL amyloid fibril structures. The fibrils are derived from the GL segments IGLV1-51*02 λ1 (FOR001, this study, PDB 7NSL) IGLV1-44*01 λ1 (FOR006, PDB 6IC3 10.2210/pdb6IC3/pdb)^21^, IGLV3-19*01 λ3 (FOR005, PDB 6Z1O 10.2210/pdb6Z1O/pdb)^23^, and IGLV6-57*02 λ6 (AL55, PDB 6HUD 10.2210/pdb6HUD/pdb)^22^. Disordered parts of the fibril proteins are indicated by dotted lines. Black: CDRs; yellow: Cys. Indigo: mutations in the CDRs and one residue before or after a CDR; magenta: mutations in framework regions; green: residues in the junctional region at the V/J interface. Red star: location of the glycosylation. **b** Sequence alignment of the four fibril proteins. CDRs are marked with gray boxes. Color coding as in (**a**). **c** Location of mutations in the corresponding, natively folded LC V_L_ domains that are based on the GL segments IGLV1-51*01 (PDB 5JZ7 10.2210/pdb5JZ7/pdb), IGLV1-44*01 (PDB 6QB6 10.2210/pdb6QB6/pdb) IGLV3-19*01 (PDB 6Q0E 10.2210/pdb6Q0E/pdb) and IGLV6-57*02 (PDB 7JVA 10.2210/pdb7JVA/pdb) CDRs are marked in black. Color coding as in (**a**).

Comparisons of the FOR001 LC with known sequences of immunoglobulin LCs and their GL segments (see “Methods” for details) revealed that the FOR001 fibril protein stems from the GL segment IGLV1-51*02. The GL precursors of the J and C segments could not be identified unambiguously. In case of the C segment, the number of residues (8, Gly111–Ser118) was too small to allow any GL assignment. In case of the J segment, IGLJ2*01, IGLJ3*01 (which are identical) and IGLJ3*02 are all possible precursors (IGLJ3*02 differs from IGLJ2*01 and IGLJ3*01 only in the first residue, which is mutated in the FOR001 protein sequence), also precluding a unique GL assignment. The FOR001 sequence shows six mutational changes compared with the GL segments IGLV1-51*02, IGLJ2*01, IGLJ3*01, and IGLJ3*02: Lys17Asn, Asn52Thr, Asn53Asp, Gly82Ala, and Asp93Glu in the V segment and Xaa99Gly in the J segment (Fig. 4a, b, indigo). The mutation Lys17Asn inserts the glycosylation site. In addition, residues Leu97–Ala98 form the variable junction between the V and J segments that arises from genetic V/J recombination (Fig. 4a, b, green).

### The location of the mutations in known fibril structures and in natively folded V_L_ domains

To identify the possible role of mutations in LC aggregation, we analyzed their position within the known fibril structures and natively folded LCs. We find that the majority of the mutations occur at solvent-exposed positions in the fibril, specifically in the FOR001 fibril. Five FOR001 mutations (Lys17Asn, Asn52Thr, Gly82Ala, Asp93Glu, and Xaa99Gly) are part of the ordered fibril core, the sixth mutation (Asn53Asp) affects a structurally disordered region (Fig. 4a, b). One residue (position 53 in the FOR001 fibril protein) is mutated in all known AL fibril structures (Fig. 4a, b) and was identified previously as a mutational hotspot in systemic AL amyloidosis^32^. It is located in the central disordered segments that are present in the FOR001, FOR005, and AL55 fibril structures (Fig. 4a), indicating that this residue does not substantially affect the fibril stability. About 79 ± 21% of the mutations of the four known AL amyloid fibrils reside within an ordered structural segment of the V_L_ domain (Supplementary Table 2). This value is identical, within error, to the percentage of residues forming the ordered fibril parts (77 ± 6%), suggesting that the mutations are not preferentially located in the fibril core. However, mutations within the ordered part of the fibril protein are generally well-accommodated in the structure and can add specific interactions. In the FOR006 fibril, they enable the interactions between Arg25 (mutated from Ser) and Glu84 [21]; in the FOR001 fibril, between Ala82 (mutated from Gly) and Leu48.

In a next step, we analyzed the position of the aggregation-prone segments in the fibril structures. These segments were identified here based on their theoretic aggregation score^21,23^. The aggregation-prone segments (as defined by an aggregation score of 5) occur within the stable core of the FOR001 fibril and other known AL amyloid fibrils (Supplementary Fig. 6a, b). By contrast, the structurally disordered regions of these fibrils typically show an aggregation score of 0 (Supplementary Fig. 6b). The fibrils with an internal disordered segment (FOR001, FOR005, and AL55) share a conserved sequence motif (PDRFSGS) with low aggregation propensity that is N-terminally preceded by a highly aggregation-prone segment (aggregation score 5, Supplementary Fig. 6a, b). In the FOR006 fibril, which does not contain an internal disordered segment, the PDRFSGS motif is not preceded by a segment with an aggregation score of 5 (Supplementary Fig. 6a, b). These data suggest that mutations and aggregation-prone segments help to define the specific fold of the observed fibril morphologies.

The mutations may additionally affect the native state, as they are found to be clustered into two regions of the globular V_L_ domains: One region is formed by the complementarity-determining region (CDR) mutations around the lower-right rim of the native fold when it is oriented as in Fig. 4c (blue spheres). This part of the structure is involved in forming the antigen-binding site and includes the shared mutated residue (position 53 in FOR001). The second region is formed by the framework mutations in the upper part of the V_L_ domain (Fig. 4c, magenta spheres). Both clusters correlate with previously described mutational hotspots of AL fibril proteins^32^. Taken together with the observations above, we conclude that the mutational changes in AL patients preferentially affect two structural sites in the native protein state. In addition, they contribute interactions to support the formation of a specific fibril morphology.

### Effect of the N-glycosylation on fibril formation

**Fig. 5:**
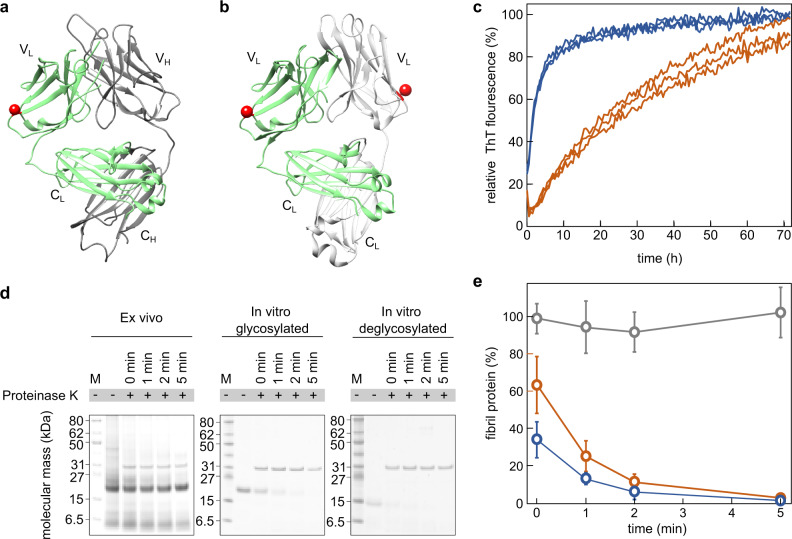
Effect of glycosylation on the formation of fibrils from FOR001 fibril protein. **a** Ribbon representations of a fragment antigen binding that contains a LC with an IGLV1-51*02 GL segment (PDB 4ODH 10.2210/pdb4ODH/pdb). The V_L_ and the C_L_ domain, as well as the variable heavy (V_H_) and constant heavy (C_H_) domains are labeled. The LC is marked green, the heavy chain is displayed in gray. Red sphere: residue homologous to the FOR001 glycosylation site. **b** Crystal structure of a LC dimer encompassing an IGLV1-51*02 GL segment (PDB 5MUD 10.2210/pdb5MUD/pdb). One LC in the dimer is marked green, the other in gray. Red sphere: as in (**a**). **c** Fibril-formation kinetics of refolded FOR001 fibril protein as obtained from real-time measurements of the ThT fluorescence intensity. Blue: deglycosylated; red: glycosylated protein. **d** Coomassie-stained denaturing-protein electrophoresis gels of samples to estimate the proteolytic stability of ex vivo FOR001 fibrils and fibrils formed in vitro from deglycosylated and glycosylated FOR001 protein. Each gel was replicated three times. M: marker. **e** Densitometric quantification of the fibril protein band (*n* = 3) of ex vivo fibrils (gray) and in vitro fibrils from deglycosylated (blue) and glycosylated FOR001 protein (red) after digestion with proteinase K for different periods of time. The band intensity of the sample before proteinase K addition was set to 100%. Based on a one-tailed Welch *t*-test, the amounts of glycosylated and deglycoslated fibril proteins differ from one another with a p-value of 0.032 and 0.049 at time points 0 min and 1 min, respectively. Error bars represent the standard deviation.

The glycosylation site is exposed on the fibril surface (Fig. 2d) and on the surface of a natively folded LC (Fig. 5a, b). In the native LC, it is far away from the interface to the heavy chain (Fig. 5a) or to the second LC in a LC dimer (Fig. 5b). These observations indicate that the glycosylation does not strongly interfere with the ability of the FOR001 LC to assemble into antibodies or LC dimers. To investigate the influence of the glycosylation on fibril formation in vitro, we purified and refolded FOR001 fibril protein from the heart. The glycosylation was removed from a fraction of the refolded protein. Both the glycosylated and the deglycosylated protein variants were able to form fibrils in vitro, as indicated by time-resolved fibrillation measurements with the amyloid-binding dye thioflavin T (ThT) (Fig. 5c). The deglycosylated FOR001 fibril protein aggregated much faster under these conditions than the glycosylated fibril protein (Fig. 5c), demonstrating that a glycosylation retards rather than accelerates fibril formation in vitro. We further investigated the proteolytic stability of the glycosylated and deglycosylated fibril samples (Fig. 5d). Both in vitro fibril samples are substantially less stable to proteolysis with proteinase K than the ex vivo fibrils, although the glycosylated in vitro fibrils seem slightly more stable to proteolysis than the deglycosylated in vitro fibrils (Fig. 5e).

## Discussion

In this study, we obtained the cryo-EM structure of a glycosylated λ1 AL amyloid fibril that was extracted from the heart tissue of patient FOR001. Adding this structure to the previously published ex vivo AL amyloid fibril structures^21–23^, there are now four known ex vivo amyloid fibril structures from systemic AL amyloidosis. Each of these structures is different (Supplementary Fig. 7). The observed differences include the precise position of the β-strands and disordered regions within the fibril protein sequence, the presence or absence of internal cavities, the topological arrangement of the secondary structural elements, the organization of the network of chemical interactions that stabilize the misfolded conformational state, and the involvement of PTMs. However, the fibrils share structural features such as a fibril core that is mainly derived from the LC V_L_ domain, the presence of an intramolecular disulfide bond, and the tertiary structures of the fibril proteins are substantially different from a natively folded LC.

In the FOR001 fibril, we identified three PTMs: a disulfide bond (Figs. 2d and 3c), a Pyro-Glu modification that affects only a fraction of the fibril proteins (Supplementary Figs. 4 and 5a, b), and a carbohydrate moiety that is linked by N-glycosylation (Supplementary Fig. 5d). The disulfide bond corresponds to the canonical disulfide of a natively folded LC V_L_ domain^5^. It occurs in all previously described cryo-EM structures of ex vivo AL amyloid fibrils^21–23^, where it connects two chain segments that present an orientation that is switched by 180° relative to the native state^21^. This disulfide has profound effects on the misfolding and on the fibril structure, as it restricts the conformational freedom of the fibril protein and leads to the requirement of a rotational switch around the disulfide bond as a key molecular event in the misfolding of amyloidogenic LCs^21^.

Pyro-Glu residues were previously observed in some^24,25^ but not all AL fibril proteins^21–23^. They also occur in N-terminally truncated Alzheimer’s Aβ peptide^33^ and may accelerate the fibrillation of this peptide in vitro^34^. In the FOR001 fibril, a significant fraction of the fibril protein lacks the Pyro-Glu modification (Supplementary Figs. 4 and 5a, b), and the modification affects a disordered region of the fibril structure (Fig. 3a). While we cannot exclude that Pryo-Glu can affect the rate of aggregation, there is, based on our data, no strong evidence to suggest that Pyro-Glu is a major driver for the generation of the specific fibril morphology observed in patient FOR001.

The third PTM is an N-glycosylation. The Asn–Xaa–Ser/Thr N-glycosylation motif occurs more frequently in AL LC sequences than in normal LCs^35,36^, and glycosylated LCs are overrepresented in systemic AL amyloidosis patients^27^, which indicates that LC glycosylation is important for systemic AL amyloidosis. However, the biophysical basis of this effect remained obscure. Several studies demonstrated that glycosylation can stabilize the native fold of globular proteins^37,38^, reduce the protein conformational dynamics^39^, or increase the solubility of proteins^37,38^, indicating that a glycosylation should render a protein less amyloidogenic. Indeed, deglycosylated FOR001 fibril protein aggregates faster than glycosylated protein, as demonstrated here by in vitro fibrillation measurements with ThT (Fig. 5c).

A potentially more important effect of glycosylation could be that it masks the amyloid deposits within the tissue, preventing their clearance by body-own mechanisms^40^. A similar amyloid-masking effect is known for serum amyloid P component^41^ that is also glycosylated. The carbohydrate of the FOR001 protein is located on the surface of the fibril structure (Fig. 2b, d) and of the natively folded LC, as indicated by analysis of the crystal structure of a homologous LC (Fig. 5a, b). Hence, the carbohydrate covers part of the surface of the fibrils or its biological precursors, consistent with a protective effect and with observations that glycosylated in vitro fibrils from FOR001 fibril protein might be slightly more stable to proteinase K digestion than in vitro formed fibrils from deglycosylated protein. However, both in vitro fibrils are much less stable to proteinase K digestion than the ex vivo FOR001 fibrils (Fig. 5d, e).

The most profound effect of glycosylation suggested by our data is that it helps to define the fibril protein fold in this patient. The fibril protein tertiary structure in the glycosylated λ1 fibril of FOR001 is significantly different from a previously described, nonglycosylated λ1 fibril^21^. The previous structure, and all known structures of nonglycosylated AL amyloid fibril, is defined by a juxtaposition of the N- and C-terminal ends of the fibril protein (Supplementary Fig. 7). In the FOR001 fibril, which carries the glycosylation in the N-terminal end of the fibril protein, no such juxtaposition is observed and the N- and C-terminal ends of the polypeptide chain are spread apart in the tertiary structure. Hence, the glycosylation may help to favor the FOR001 fibril protein fold by preventing the association of the N- and C-terminal ends of the fibril protein.

Several previous observations have suggested that the fibril protein fold and the fibril morphology are crucial for the pathological process of an amyloid disease. Consistent fibril protein folds and fibril morphologies can be found in different patients or animals that are affected by the same disease variant and allelic variant of the fibril precursor protein^42–44^. Different fibril protein structures or fibril morphologies are associated with different disease variants^44–46^. Ex vivo fibril morphologies are often different from fibrils formed in vitro from the same proteins^20,23,44,47–49^ and more protease-stable than their in vitro formed counterparts^47,49^. In particular, the latter observations led to the hypothesis that pathogenic amyloid fibril structures may have been selected inside the body by their high proteolytic resistance and ability to escape endogenous clearance systems^49,50^. The present observations further support this view as they imply that the disulfide formation, the glycosylation, and the mutational changes inserted during somatic hypermutation helped to shape the specific fibril morphology that is associated with patient FOR001 and which shows a high protease stability (Fig. 5d, e).

In summary, we have shown that in AL amyloidosis, fibril deposits can interfere with the physiological function of heart tissue by disrupting its ordered architecture. PTMs and the specific mutational changes that characterize amyloidogenic LCs facilitate the formation of patient-specific fibril morphologies that are able to survive under the otherwise hostile and proteolytic conditions inside the body. The resulting fibrils are thus able to accumulate, proliferate, and cause damage to the surrounding tissue. The differences in the available cryo-EM structures of fibrils from AL amyloidosis patients underline the patient-specific nature of the disease and make clear the need for investigating AL fibril structures from many patient cases in order to identify common principles of AL amyloid formation.

## Methods

### Source of the fibril-containing tissue

The fibrils were extracted from the explanted heart of a male patient (FOR001), who suffered from systemic AL amyloidosis with cardiac involvement and underwent cardiac surgery at the age of 51 years. The underlying condition was a monoclonal gammopathy. The patient was treated within the heart-transplant program of the University Hospital Heidelberg. The explanted heart tissue was stored at −80 °C. The study was approved by the ethical committees of the University of Heidelberg (123/2006) and of Ulm University (203/18). Informed consent was obtained from the patient for the analysis of the amyloid deposits.

### Visualization of amyloid fibril deposits in heart tissue using STEM and SEM

Frozen pieces of FOR001 heart tissue (~1 mm^3^) were fixed in a solution of 0.1% (w/v) glutaraldehyde, 4% (w/v) paraformaldehyde, and 1% (w/v) saccharose in 0.1 M sodium phosphate buffer (pH 7.3) overnight at 4 °C. The further sample preparation for STEM and SEM was based on a protocol from a previous publication^51^. In brief, the fixed tissue pieces were cut into ~200-μm slices with a scalpel and then high-pressure frozen with a Compact 01 high-pressure freezing device (Engineering Office M. Wohlwend) and freeze-substituted in a medium consisting of 0.1% (w/v) uranyl acetate, 0.2% (w/v) osmium tetroxide, and 5% (v/v) water in acetone using an AFS2 freeze-substitution device (Leica Microsystems) with which the temperature was raised from −90 °C to room temperature over a period of 19 h. Afterward, the tissue pieces were embedded in epoxy resin (Sigma-Aldrich) starting with a mixture of 30% (v/v) resin in acetone for 1 h followed by 60% (v/v) resin in acetone for 3 h and 100% resin overnight and polymerization in fresh 100% resin by incubation at 60 °C for 48 h. For SEM imaging, 200-nm thin sections were cut from the polymerized samples using the ultramicrotome Ultracut (Leica Microsystems) equipped with a 45° diamond knife (Diatome). Sections were mounted on glow-discharged silicon wafers and stained with a 0.3% (w/v) lead citrate solution in water for 1 min, washed with distilled water, dried, and imaged in a Hitachi S-5200 field emission scanning electron microscope, detecting the secondary electron signal at 5 kV in analysis mode. For STEM, 300-nm thin sections were cut with a 45° diamond knife and processed in a similar way as described in a previous publication^52^. In brief, sections were mounted on copper grids with 200 parallel grid bars (Plano). After attachment of 15-nm colloidal gold fiducials (Aurion), sections were coated with carbon by electron-beam evaporation in a Baf 300 (BalTec). Tomograms were acquired with a STEM JEM-2100F (JEOL) operated at 200 kV. Tilt series were acquired from −72° to +72° with a 1.5° increment using the bright-field detector. The pixel size was 1.395 nm. Tilt series were reconstructed to tomograms by weighted back projection using an emulated simultaneous iterative-reconstruction technique-like filter (20 iterations) and segmentation of fibrils and cell membranes was performed with the IMOD software package^53^, version 4.9.0.

### Measurement of the persistence length

A total of 197 amyloid fibrils in heart tissue were traced in the STEM tomograms and were then available as polygonal chains. Based on this representation, the squared end-to-end distance *R*, i.e., the Cartesian plane distance between the starting and end points, and the contour length *L,* i.e., the sum of the length of all line segments of the polygonal chain, were computed for each fibril. The persistence length *P* of the fibrils was then determined by regression analysis using the formula

1 $${R}^{2}=2{PL}\left(1-\frac{P}{L}\left(1-{e}^{\frac{-L}{P}}\right)\right)$$

according to Kollmer et al.^54^ and using the curve-fitting tool in Matlab (MATLAB 2019, The MathWorks). *P* is contained in the confidence interval from 0.66 μm to 0.81 μm in 95% of all cases. The bending rigidity *B* is computed based on *P* according to the formula

2 $$B={k}_{B}{TP}$$

where *k*_B_ and *T* denote the Boltzmann constant and the temperature (300 K), respectively^21,55^.

### Fibril extraction from FOR001 heart tissue

Fibrils were extracted from FOR001 heart tissue using a previously established protocol^56^. In brief, 250 mg of heart tissue was diced with a scalpel and washed five times with Tris Calcium Buffer (TCB) [20 mM Tris, 138 mM NaCl, 2 mM CaCl_2_, and 0.1% (w/v) NaN_3_, pH 8.0]. Each washing step consisted of the addition of 0.5 mL of ice-cold TCB to the pellet, the homogenization of the tissue with a Kontes Pellet Pestle, and the separation of the tissue from the supernatant by centrifugation for 5 min at 3100 × *g* and 4 °C, followed by the removal of the supernatant. The tissue pellet after the fifth washing step was resuspended in 1 mL of TCB containing 5 mg/mL *Clostridium histolyticum* collagenase (Sigma-Aldrich) and one tablet of complete ethylenediaminetetraacetic acid (EDTA)-free protease-inhibitor cocktail (Roche) per 7 mL of TCB. After an overnight incubation of the digest at 37 °C in a horizontal orbital shaker (750 rpm), the sample was centrifuged for 30 min at 3100 × *g* and 4 °C. The pellet was subjected to ten washing steps which were performed in the same manner as the TCB washing steps described above, except that 0.5 mL of Tris EDTA buffer [20 mM Tris, 140 mM NaCl, 10 mM EDTA, and 0.1% (w/v) NaN_3_, pH 8.0] was used instead of TCB. The pellet from the last wash was resuspended in 0.5 mL of ice-cold distilled water, mixed with a pipette, and centrifuged for 5 min at 3100 × *g* and 4 °C. The fibril-containing supernatant was retained and the pellet was submitted to nine more cycles of resuspension in water and centrifugation. The supernatants were retained to check for the presence of fibrils.

### Platinum side-shadowing and TEM

The handedness of the fibrils was determined by platinum side-shadowing and TEM. Formvar and carbon-coated 200 mesh copper grids (Plano) were glow discharged for 40 s at 40 mA using a PELCO easiGlow glow-discharge cleaning system (Ted Pella). About 15 μL of the fibril solution were applied to the grid and incubated for 30 s at room temperature. The grid was blotted using filter paper (Whatman) to remove excess fluid. The grid was washed three times with 10 μL of distilled water and dried at room temperature. Platinum was evaporated at an angle of 30° onto the grid to form a 1-nm-thick layer by use of a Balzers BAF 300 coating device. Grids were imaged using a JEM-1400 TEM (JEOL) that was operated at an acceleration voltage of 120 kV. The images were recorded with an F216 camera (TVIPS).

### Cryo-EM sample preparation and data collection

C-flat 1.2/1.3 400-mesh holey carbon-coated grids (Science Services) were glow-discharged at 40 mA for 40 s using a PELCO easiGlow glow-discharge cleaning system (Ted Pella). Conditions of grid preparation were optimized with the help of a Vitrobot Mark 3 (Thermo Fisher Scientific) and checked in a 200-kV JEM 2100 F transmission electron microscope (JEOL) that was equipped with a DE12 detector (Direct Electron). The grids for data collection were prepared by application of 3.5 μL of fibril solution to a grid, incubation for 30 s at >95% humidity, both-side blotting using filter paper (Whatman), and plunging into liquid ethane (~103 K). The data set was recorded with a Titan Krios transmission electron microscope (Thermo Fisher Scientific) at 300 kV and applying a Gatan imaging filter with a 20-eV slit. The images were recorded with a K2-Summit detector (Gatan) in counting mode. The software SerialEM v3.7 was used for data collection. In total, 3033 micrographs were collected from a single grid. See Supplementary Table 1 for further details. Global fibril parameters, such as width and crossover distance, were measured using Fiji v1.52^57^. The proportion of fibrils showing the reconstructed morphology was determined by analyzing all fibrils (length at least 200 nm) in 100 micrographs.

### Reconstruction of the 3D map

Motion correction and dose-weighting was carried out with MotionCor2^58^. For predicting, refining, and correction of the contrast transfer function, Gctf v1.06^59^ was used. Helical reconstruction was performed using Relion 3.0^60^. As a first step, 279,338 particles were picked manually with a box size of ~312 Å and an interbox distance of 34.6 Å (~11%). After a reference-free 2D classification using 279 classes and a regularization value of T = 2 in the first run, another 2D classification was performed with 50 classes. 2D classes were selected based on the visibility of a z-axial repeat at ~4.8 Å and bad classes were excluded. An initial 3D model was generated using Relion’s initial-model job. To generate a better reference for 3D classification, fibrils with visible crossovers were picked from 28 micrographs, resulting in 244 particles. These particles were subjected to a 3D classification with the initial model as a reference. This treatment resulted in a map with fibril-like features. Using this map as a reference, the particle set selected from 2D classification was subjected to several rounds of 3D classification, 3D refinement, and post-processing. The resolution of the resulting map was estimated to be 3.6 Å. To obtain a more homogeneous set of particles, fibrils were picked more selectively, yielding 43,308 particles with a box size of ~270 Å. Using the previously obtained post-processed map as a reference (with adjusted box size), subsequent steps of 3D classification, 3D refinement, and post-processing yielded a map at a resolution of 3.4 Å. All manually picked particles were retained in this reconstruction. Further improvement of map resolution was accomplished through Bayesian polishing, resulting in a final map resolution of 3.1 Å, based on the value of the FSC curve for two independently refined half-maps at 0.143. An estimated map-sharpening B-factor of −67.383 Å^2^ was applied. The map had a twist of −1.45566° and a rise of 4.76311 Å. These values agree with the twist value calculated from the measured crossover distances on the cryo-EM micrographs, and with the rise value measured from the micrograph power spectra.

### Model building and refinement

The software Coot^61^ v0.8.9 was used to manually build the protein model de novo. First, the 3D map was traced and a poly-L-Ala chain created representing the protein backbone. The residues of this chain were then mutated to the FOR001 fibril protein sequence as determined by MS. This initial model was then further improved by subsequent rounds of manual and automated refinement (phenix.real_space_refine)^62^ as implemented in Phenix v1.16^63^. Noncrystallographic symmetry and secondary-structure restraints were imposed. Model-based automated sharpening of the map (phenix.auto_sharpen)^63^ yielded an improved map, which was used to further refine the model. The quality of the model was assessed using the MolProbity^64^-generated validation report. Modeling parameters are listed in Supplementary Table 1.

### Protein sequence determination by electrospray-ionization MS

About 2 μg of refolded, lyophilized and glycosylated fibril protein was resuspended in 15 μL of buffer [280 mM Tris/HCl pH 6.8, 9% (w/v) sodium dodecyl sulfate (SDS), 33.3% (w/v) glycerol, and 100 mM dithiothreitol] and processed by denaturing protein gel electrophoresis. Afterward, the gel band of the fibril protein was cut out and washed by a 10-min incubation in the respective protease buffer (see below), and subsequently, in a mixture of 50% (v/v) protease buffer and 50% (v/v) ACN for 10 min. These incubation steps were repeated twice, followed by vacuum drying. Dried gel slices were reduced with 5 mM dithiothreitol (AppliChem) in 50 mM ammonium bicarbonate, pH 8.0, for 20 min at room temperature and subsequently alkylated with 55 mM iodoacetamide (Sigma-Aldrich) in 10 mM ammonium bicarbonate for 20 min at 37 °C. The gel slices were placed in five different protease solutions (trypsin in 50 mM ammonium bicarbonate, pH 8.0; LysC in 50 mM ammonium bicarbonate, pH 8.0; elastase in 50 mM Tris/HCl, pH 9.0; chymotrypsin in 50 mM Tris/HCl buffer, pH 8.0, 10 mM CaCl_2_; pepsin 40 mM HCl, pH 1.5). Each protease was used at 0.33 ng/μL concentration and digestion was carried out overnight at 37 °C (except for chymotrypsin at 25 °C). The resulting peptides were released from the gel slices in two steps: the first step was to add 20 μL of a solution containing 50% (v/v) ACN and 0.1% (v/v) TFA; the second step was an incubation in an ultrasonic bath (Bandelin Sonorex Super 10 P) at 100% intensity for 10 min each. ACN was evaporated and samples were filled to 15 μL with 0.1% TFA (v/v).

Samples were separated by liquid chromatography using a U3000 RSLCnano (Thermo Fisher Scientific) online coupled to the mass spectrometer with an Acclaim PepMap analytical column (75 μm × 500 mm, 2 μm, 100-Å pore size, Thermo Fisher Scientific) in combination with a C18 μ-precolumn (0.3 mm × 5 mm, PepMap, Dionex LC Packings, Thermo Fisher Scientific). First, samples were washed with 0.1% (v/v) TFA for 5 min at a flow rate of 30 μL/min. Subsequent separation was carried out employing a flow rate of 250 nL/min using a gradient consisting of solvent A [0.1% (v/v) formic acid] and solvent B [86% (v/v) ACN, 0.1% (v/v) formic acid]. The main column was initially equilibrated in a mixture containing 5% (v/v) solvent B and 95% (v/v) solvent A. For elution, the percentage of solvent B was raised from 5 to 15% over a period of 10 min, followed by an increase from 15 to 40% over 20 min. Fractions from the main column directly eluted into the ionization module and were further analyzed by MS.

Samples were measured using an LTQ Orbitrap Velos Pro system (Thermo Fisher Scientific). The mass spectrometer was equipped with a nanoelectrospray ion source and distal-coated SilicaTips (FS360-20-10-D, New Objective). The instrument was externally calibrated using standard compounds (LTQ Velos ESI Positive Ion Calibration Solution, Pierce, Thermo Scientific). The system was operated using the following parameters: spray voltage, 1.5 kV; capillary temperature, 250 °C; S-lens radio-frequency level, 68.9%. The software XCalibur 2.2 SP1.48 (Thermo Fisher Scientific) was used for data-dependent MS/MS analyses. Full scans ranging from mass-to-charge ratio (m/z) 370–1700 were acquired in the Orbitrap at a resolution of 30,000 (at m/z 400) with automatic gain control enabled and set to 10^6^ ions and a maximum fill time of 500 ms. Collision-induced dissociation was employed as the fragmentation method on individual sample sets. Per survey scan, 10 ions were selected. Single charged ions were rejected and the m/z peaks of fragmented single-charged ions were excluded from fragmentation for 60 s. In the linear ion trap, the automatic gain control was set to 10,000 ions and a maximum fill time of 100 ms. For MS/MS fragmentation, a normalized collision energy of 35% with an ‘activation q’ of 0.25 and an activation time of 30 ms was used. The resulting fragments were analyzed using the linear ion-trap part at rapid scan speeds. Subsequent detection of fragmentation spectra was performed in the Orbitrap mass analyzer at a resolution of 7500.

For de novo sequencing, the Peaks AB Software (Bioinformatics Solutions) was used. The resulting sequence was then used as a target for further analyses using the Peaks X software suite (Bioinformatics Solutions) in order to confirm the de novo sequence. For all analyses, the mass accuracy was set to 10 ppm on intact peptide masses and 0.5 Da. Various PTMs were considered, including the deamidation of Asn or Gln residues, pyroglutamate modifications (Gln), oxidation of Met, as well as carbamidomethylated Cys as a result of the alkylation. Ile/Leu as well as Gln/Lys have the same molecular weights and could not be uniquely determined. These residues were assigned based on homology considerations.

### MS analysis of the total fibril protein mass

Lyophilized FOR001 fibril protein was resuspended in Glycoprotein Denaturing Buffer (New England Biolabs) and subsequently it was deglycosylated without the heating step of the standard protocol. The deglycosylated protein was diluted with 0.1% TFA (v/v) to obtain a concentration of 66 μg/mL. It was applied with a flow rate of 10 μl/min onto a PepSwift trap column (200 μm × 5 mm, Thermo Fisher Scientific) in combination with a monolithic ProSwift RP-4H analytical column (100 μm × 50 cm, Thermo Fisher Scientific), which was connected to a U3000 RSLCnano (Thermo Fisher Scientific) that was coupled to the mass spectrometer. The fibril protein was eluted using a gradient of solvent B [86% (v/v) ACN, 0.1% (v/v) formic acid] and solvent A [0.1% (v/v) formic acid] with a flow rate of 1 μL/min. The gradient started with an increase of 5–55% solvent B over a period of 75 min, followed by an increase from 55 to 95% over 15 min. The concentration of 95% solvent B stayed constant for 3 minutes with a subsequent reduction from 95 to 5% solvent B over 9 min. Fractions from the ProSwift RP-4H analytical column directly eluted into the ionization module and were further analyzed by MS. Samples were measured using an LTQ Orbitrap Elite system (Thermo Fisher Scientific). The mass spectrometer was equipped with a nanoelectrospray ion source and distal-coated SilicaTips (FS360-20-10-D, New Objective). The instrument was externally calibrated using standard compounds (LTQ Velos ESI Positive Ion Calibration Solution, Pierce, Thermo Scientific) and operated using the following parameters: spray voltage, 1.5 kV; capillary temperature, 250 °C; S-lens radio-frequency level, 68.9%. The software XCalibur 2.2 SP1.48 (Thermo Fisher Scientific) was used for data-dependent MS/MS analyses. Full scans ranging from mass-to-charge ratio (m/z) 370–1700 were acquired in the Orbitrap at a resolution of 30,000 (at m/z 400) with automatic gain control enabled and set to 10^6^ ions and a maximum fill time of 500 ms. The raw data were deconvoluted by the MASH Explorer^65^ using default settings and the Quick Deconvolution feature. All calculated monoisotopic masses with a score equal to or above 94% resulting from initial m/z peaks with 5 charges or more were considered as correct and are shown in Supplementary Fig. 5b, c. The deconvoluted mass peaks were further assigned to protein species by using the software mMass^66^ considering a tolerance of 0.1 Da and a peak charge of 0. Sequence modifications were set as follows: pyroglutamylation at Gln1 was set as variable, whereas the disulfide bond between Cys22 and Cys89 was set as fixed.

### Refolding of the FOR001 fibril protein

Solid guanidine hydrochloride was added to a sample of ex vivo FOR001 fibrils to reach a final concentration of 6 M followed by an overnight incubation at room temperature to disaggregate the fibrils. The protein was refolded by dialysis (molecular weight cutoff 3.5 kDa, Spectra/Por 6 Dialysis Membrane Pre-wetted RC Tubing, Spectrum Labs) against 20 mM Tris buffer, pH 8.0, for 24 h at 4 °C. The protein was purified by anion-exchange chromatography with Q-SepharoseFF medium (10 mL, Cytiva) in an XK 16/20 column (Cytiva) with a slope gradient from 0% to 100% elution buffer [20 mM Tris buffer, 1 M NaCl, pH 8.0] over 20 column volumes (CVs). The fibril protein-containing fractions, as identified by protein-gel electrophoresis, were purified further with a Resource 15 RPC column (3 mL, Cytiva) that was equilibrated in solvent A [0.1% (v/v) trifluoroacetic acid (TFA) in water]. The protein was eluted through a slope gradient from 0 to 58% solvent B [86% (v/v) acetonitrile (ACN), 0.1% (v/v) TFA] over 20 CVs, followed by second gradient from 58 to 100% solvent B over 4 CVs to remove other bound proteins. Fractions were collected and the fractions containing the FOR001 fibril protein were identified by protein gel electrophoresis, pooled, and lyophilized.

### Deglycosylation of the refolded FOR001 fibril protein

For the experiment shown in Supplementary Fig. 5a, the lyophilized, refolded FOR001 fibril protein was dissolved in water at approximately 2 mg/mL concentration. The exact protein concentration was determined by the intrinsic protein absorbance at 280 nm. This solution was mixed with water and SDS-containing 10 × glycoprotein-denaturing buffer [5% (w/v) SDS, 400 mM dithiolthreitol] (New England Biolabs) to generate a final FOR001 fibril protein solution with the volume *V*_Prot_ containing a protein concentration of 1 mg/mL and 1 x Glycoprotein Denaturing Buffer (New England Biolabs). The protein was heated for 10 min at 100 °C to partially denature the protein before it was cooled to room temperature. For N-deglycosylation, the heated sample was mixed with 1/5 *V*_Prot_ of a 10% (v/v) solution of the detergent Nonident P40 in water (New England Biolabs), 1/5 *V*_Prot_ 10 × Glycobuffer 2 [500 mM sodium phosphate, pH 7.5] (New England Biolabs), 1/2 *V*_Prot_ water, and 1/10 *V*_Prot_ PNGase F in 20 mM Tris/HCl, 50 mM NaCl, and 5 mM EDTA, pH 7.5 (New England Biolabs), and incubated for 1 h at 37 °C. For O-deglycosylation, the heated sample was mixed with 1/5 *V*_Prot_ of a 10% (v/v) Nonident P40 solution (New England Biolabs), 1/5 *V*_Prot_ 10 × Glycobuffer 2 (New England Biolabs), 3/10 *V*_Prot_ water, 1/10 *V*_Prot_ O-glycosidase in 20 mM Tris/HCl, 50 mM NaCl, and 1 mM EDTA, pH 7.5 (New England Biolabs), and 1/5 *V*_Prot_ neuraminidase in 20 mM Tris/HCl, 50 mM NaCl, and 5 mM EDTA, pH 7.5 (New England Biolabs), and incubated for 10 min at 37 °C. The deglycosylation was checked by denaturing protein-gel electrophoresis. N- and O-glycosylated fetuin protein, which we purchased as a 10 mg/mL solution from New England Biolabs, was used as a control substance. For the experiments reported in Fig. 5, the lyophilized, refolded FOR001 protein was dissolved in water at 1 mg/mL concentration and denatured—without SDS and dithiothreitol—for 10 min at 100 °C, as the SDS in the denaturing buffer was found to interfere with the subsequent reversed-phase chromatography.

### Protein-concentration measurement based on the intrinsic absorbance at 280 nm

About 40 μL of protein solution was mixed with 160 μL of 7.5 M guanidine hydrochloride (Carl Roth) in 25 mM sodium phosphate buffer, pH 6.5. The absorbance was measured at 280 nm in a Lambda Bio+ ultraviolet/visible (PerkinElmer) spectrometer using a Quartz Suprasil R Ultra-Micro cuvette (Hellma). The protein concentration was determined based on the Lambert–Beer law using a theoretic molar extinction coefficient of 16,740 M^−1^ cm^−1^ for the FOR001 fibril protein according to the method of Gill and von Hippel^67^.

### Fibril-formation kinetics measurements using ThT

Refolded and lyophilized FOR001 fibril protein (glycoslylated or deglycosylated) was dissolved at 2 mg/mL concentration in water. Aggregation kinetics measurements were set up in PF 96-well F-bottom black microplates (Greiner Bio-One International). Each well was filled with 100 μL of sample, containing 0.4 mg/mL glycosylated or deglycosylated fibril protein, 20 μM ThT and 10 mM sodium acetate, 10 mM boric acid, 10 mM sodium citrate, pH 4.0, and 150 mM NaCl. The plates were sealed with Rotilabo-sealing film (Carl Roth) and incubated at 37 °C in FLUOstar Omega (BMG Labtech) for 72 h. During incubation, the plates were agitated by orbital shaking at 300 rpm, which was paused during measurement. The fluorescence emission intensity at 490 nm was recorded every 30 min upon excitation at 450 nm.

### Proteinase K digestion of amyloid fibrils

Aliquots of solutions containing ex vivo FOR001 amyloid fibrils or in vitro formed fibrils from glycosylated or deglycosylated FOR001 fibril protein (from the ThT kinetic experiment) were mixed with water and a 10 × buffer stock [200 mM Tris, pH 8.0, 1.4 M NaCl, 20 mM CaCl_2_, and 1% (w/v) NaN_3_] to reach a total volume of 60 μL containing 0.2 mg/mL protein in 1 × buffer. A first aliquot (10 μL) was withdrawn from this solution and retained for gel electrophoresis as the control sample without protease. The remaining 50 μL of the protein solution were mixed with 1 μL of a 2 mg/mL proteinase K solution (Thermo Fisher Scientific). Immediately afterward, a second aliquot (10 μL) was removed (0-min sample). The remaining solution was incubated at 37 °C in a heating block and further aliquots (10 μL) were withdrawn after 1 min, 2 min, and 5 min. As soon as an aliquot was withdrawn, it was mixed with 1 μL of 200 mM phenylmethylsulfonyl fluoride (PMSF) (Carl Roth) in methanol, incubated for 1 min at room temperature, and flash-frozen in liquid nitrogen. After the experiment, all aliquots were brought to room temperature and analyzed by denaturing protein-gel electrophoresis. The resulting protein bands (72 × 150 pixels) were densitometrically quantified using the program Fiji v1.52^57^. The intensity of the fibril protein band without proteinase K was set to 100%, and an equally sized area on the gel without protein at 0%.

### Denaturing protein gel electrophoresis

Samples from the deglycosylation experiment and proteolytic stability measurement (10-μL volume for deglycosylation, 11-μL volume for proteolytic stability, including 1-μL PMSF) were mixed with 2 μL of 10 × NuPAGE reducing agent (Thermo Fisher Scientific), 5 μL of 4 × NuPAGE LDS sample buffer (Thermo Fisher Scientific), and water to generate a sample with a total volume of 20 μL. The solution was heated at 95 °C for 10 min and applied onto a 4–12% NuPAGE Bis-Tris gel (Thermo Fisher Scientific), operated in NuPAGE MES SDS running buffer (Thermo Fisher Scientific). BlueEasy Prestained (Genetics) was used as a marker. The gel was stained in a solution containing 30% (v/v) ethanol, 10% (v/v) acetic acid, and 0.25% (w/v) Coomassie brilliant blue and destained with a solution containing 20% (v/v) ethanol and 10% (v/v) acetic acid.

### Analysis of GL segments and mutations

The FOR001 amino acid sequence was used to search the IgBLAST database (https://www.ncbi.nlm.nih.gov/igblast/), which returned the GL segment IGLV1-51*02. The protein sequence of the GL segment was retrieved from the VBase2 database (http://www.vbase2.org/). The FOR001 J segment was compared with the five functional *IGLJ* GL segments in the GenBank database (https://www.ncbi.nlm.nih.gov/genbank/). It matched both *IGLJ2* (Gene ID: 28832) and *IGLJ3* (GeneID: 28831) and no unique source could be determined. Nor could we identify the GL precursor of the C segment as most of the C_L_ domain is missing in the FOR001 fibril protein. The residues Leu97–Ala98 constitute the variable V/J junctional region. CDRs were determined with abYsis (http://www.abysis.org/abysis/) based on the Kabat definition^68^. In this paper, all mutations are represented in the direction GL to fibril protein.

### Computation of the aggregation score

To compute the aggregation score, we used TANGO version 2.1^69^, WALTZ^70^, FoldAmyloid^71^, Aggrescan^72^, and PASTA2.0^73^. These programs calculate a residue-specific aggregation potential. The following settings were chosen for each program: TANGO: temperature: 309.15 K, ionic strength: 0.02 M, and concentration: 1 M and pH 7.0; WALTZ: the threshold was set to high sensitivity and the pH to 7.0; FoldAmyloid: scale: triple hybrid, averaging frame: 5; Aggrescan: default settings; PASTA: 90% specificity, top pairing energies: 22. Residues with a high aggregation potential are defined as follows: TANGO: β-sheet aggregation values above 0.0; WALTZ: total sequence score above 0.0; Foldamyloid: five successive amino acids with a triple-hybrid threshold above 0.062; Aggrescan: aggregation-propensity values above −0.02; PASTA: PASTA energy units below −2.8. An aggregation score of 0 means that none of the programs identifies a high aggregation score for a given residue. An aggregation score of 5 means that all five programs predict a high aggregation score for that residue.

### Protein structure representation

The images of the density map and protein model were created with the software UCSF Chimera v1.14^74^. Hydrogen bonds were defined according to the criteria of both UCSF Chimera v.1.14^74^ and the software Coot v0.8.9^61^. The β-sheets were defined as a minimum of two residues having φ/ψ angles in the β-sheet region of the Ramachandran plot and at least one-backbone hydrogen bond.

### Sample statistics

In this paper errors report the standard deviation. In Fig. 5e, a one-tailed Welch *t*-test was used.

### Reporting summary

Further information on research design is available in the Nature Research Reporting Summary linked to this article.

## Supplementary information

Supplementary Information

Reporting Summary

## Data Availability

The datasets used during the current study are available from public repositories and/or from the corresponding author on reasonable request. The cryo-EM map of the FOR001 fibrils was deposited in the Electron Microscopy Data Bank (https://www.ebi.ac.uk/pdbe/emdb/) with the accession code EMD-12570. The coordinates of the corresponding atomic model were deposited in the PDB (https://www.rcsb.org/) under the accession code 7NSL. The cryo-EM data of the FOR001 fibrils were deposited on EMPIAR (https://www.ebi.ac.uk/pdbe/emdb/empiar/) with the accession code EMPIAR-10730. The following published PDB structures were used in the paper: 4ODH, 6IC3, 6Z1O, 6HUD, 5JZ7, 6QB6, 6Q0E, 7JVA, and 5MUD. The accession codes for the *IGLV1-51**02 segment from the IMGT database are M30446 and from the VBase2 humIGLV015. The *IGLJ2* and *IGLJ3* gene segments were taken from GenBank with the Gene IDs 28832 and 28831, respectively. Source data are provided with this paper for the following figures: Fig. 1c, Fig. 5c, d, e, Supplementary Fig. 1a, Supplementary Fig. 2, Supplementary Fig. 5d, and Supplementary Fig. 6. Source data are provided with this paper.

## Source data

Source Data

## References

[1] Blancas-Mejia LM (2018). Immunoglobulin light chain amyloid aggregation. Chem. Commun..

[2] Herrera, G. A., Teng, J., Turbat-Herrera, E. A., Zeng, C. & del Pozo-Yauner, L. Understanding Mesangial Pathobiology in AL-amyloidosis and Monoclonal Immunoglobulin Light Chain Deposition Disease. *Kidney Int. Rep.* (2020). 10.1016/j.ekir.2020.07.013 PMC7609979 33163710

[3] Wechalekar AD (2013). A European collaborative study of treatment outcomes in 346 patients with cardiac stage III AL amyloidosis. Blood.

[4] Merlini G (2018). Systemic immunoglobulin light chain amyloidosis. Nat. Rev. Dis. Prim..

[5] Janeway C. A. Jr., Travers, P., Walport, M. & Shlomchik, M. J. *Immunobiology: the Immune System in Health and Disease*. 5th edn (Garland Science, 2001). The generation of diversity in immunoglobulins.

[6] Lefranc MP (2001). IMGT®, the international ImMunoGeneTics database. Nucl. Acids Res..

[7] Comenzo RL, Zhang Y, Martinez C, Osman K, Herrera GA (2001). The tropism of organ involvement in primary systemic amyloidosis: contributions of Ig VL germ line gene use and clonal plasma cell burden. Blood.

[8] Perfetti V (2002). Analysis of Vλ-Jλ expression in plasma cells from primary (AL) amyloidosis and normal bone marrow identifies 3r (λIII) as a new amyloid-associated germline gene segment. Blood.

[9] Abraham RS (2003). Immunoglobulin light chain variable (V) region genes influence clinical presentation and outcome in light chain–associated amyloidosis (AL). Blood.

[10] Kourelis TV (2017). Clarifying immunoglobulin gene usage in systemic and localized immunoglobulin light-chain amyloidosis by mass spectrometry. Blood.

[11] Perfetti V (2012). The repertoire of lambda light chains causing predominant amyloid heart involvement and identification of a preferentially involved germline gene, IGLV1-44. Blood.

[12] Hurle MR, Helms LR, Li LIN, Chan W, Wetzel R (1994). A role for destabilizing amino acid replacements in light-chain amyloidosis. Proc. Natl. Acad. Sci. USA.

[13] Wall, J. et al. Thermodynamic instability of human lambda 6 light chains: Correlation with fibrillogenicity Biochemistry. *Biochem.* **38**, 14101–14108 (1999). 10.1021/bi991131j 10529258

[14] Oberti L (2017). Concurrent structural and biophysical traits link with immunoglobulin light chains amyloid propensity. Sci. Rep..

[15] Kazman P (2020). Fatal amyloid formation in a patient’s antibody light chain is caused by a single point mutation. eLife.

[16] Rottenaicher GJ (2021). Molecular mechanism of amyloidogenic mutations in hypervariable regions of antibody light chains. J. Biol. Chem..

[17] Blancas-Mejía LM (2014). Kinetic control in protein folding for light chain amyloidosis and the differential effects of somatic mutations. J. Mol. Biol..

[18] Piehl DW, Blancas-Mejía LM, Ramirez-Alvarado M, Rienstra CM (2017). Solid-state NMR chemical shift assignments for AL-09 V L immunoglobulin light chain fibrils. Biomol. NMR Assign..

[19] Pradhan T (2020). Seeded fibrils of the germline variant of human λ-III immunoglobulin light chain FOR005 have a similar core as patient fibrils with reduced stability. J. Biol. Chem..

[20] Annamalai K (2017). Common fibril structures imply systemically conserved protein misfolding pathways in vivo. Angew. Chem..

[21] Radamaker L (2019). Cryo-EM structure of a light chain-derived amyloid fibril from a patient with systemic AL amyloidosis. Nat. Commun..

[22] Swuec P (2019). Cryo-EM structure of cardiac amyloid fibrils from an immunoglobulin light chain AL amyloidosis patient. Nat. Commun..

[23] Radamaker L (2021). Cryo-EM reveals structural breaks in a patient-derived amyloid fibril from systemic AL amyloidosis. Nat. Commun..

[24] Yazaki M, Liepnieks JJ, Callaghan J, Connolly CE, Benson MD (2004). Chemical characterization of a lambda I amyloid protein isolated from formalin-fixed and paraffin-embedded tissue sections. Amyloid.

[25] Lu Y, Jiang Y, Prokaeva T, Connors LH, Costello CE (2017). Oxidative post-translational modifications of an amyloidogenic immunoglobulin light chain protein. Int. J. Mass Spectrom..

[26] Kumar S (2019). Assay to rapidly screen for immunoglobulin light chain glycosylation: a potential path to earlier AL diagnosis for a subset of patients. Leukemia.

[27] Mellors PW (2021). MASS-FIX for the detection of monoclonal proteins and light chain N-glycosylation in routine clinical practice: a cross-sectional study of 6315 patients. Blood Cancer J..

[28] Dispenzieri A (2020). N-glycosylation of monoclonal light chains on routine MASS-FIX testing is a risk factor for MGUS progression. Leukemia.

[29] Sagis LM, Veerman C, van der Linden E (2004). Mesoscopic properties of semiflexible amyloid fibrils. Langmuir.

[30] Adamcik J, Mezzenga R (2012). Study of amyloid fibrils via atomic force microscopy. Curr. Opin. Colloid Interface Sci..

[31] Palladini G (2006). Circulating amyloidogenic free light chains and serum N-terminal natriuretic peptide type B decrease simultaneously in association with improvement of survival in AL. Blood.

[32] Poshusta TL (2009). Mutations in specific structural regions of immunoglobulin light chains are associated with free light chain levels in patients with AL amyloidosis. PloS ONE.

[33] Thal DR, Walter J, Saido TC, Fändrich M (2015). Neuropathology and biochemistry of Aβ and its aggregates in Alzheimer’s disease. Acta Neuropathol..

[34] Wulff M (2016). Enhanced fibril fragmentation of N-terminally truncated and pyroglutamyl-modified Aβ peptides. Angew. Chem. Int. Ed..

[35] Stevens FJ (2000). Four structural risk factors identify most fibril-forming kappa light chains. Amyloid.

[36] Omtvedt LA (2000). Glycosylation of immunoglobulin light chains associated with amyloidosis. Amyloid.

[37] Solá RJ, Griebenow KAI (2009). Effects of glycosylation on the stability of protein pharmaceuticals. J. Pharm. Sci..

[38] Schwarz F, Aebi M (2011). Mechanisms and principles of N-linked protein glycosylation. Curr. Opin. Struct. Biol..

[39] Lee HS, Qi Y, Im W (2015). Effects of N-glycosylation on protein conformation and dynamics: Protein Data Bank analysis and molecular dynamics simulation study. Sci. Rep..

[40] Bellotti V, Mangione P, Merlini G (2000). Immunoglobulin light chain amyloidosis—the archetype of structural and pathogenic variability. J. Struct. Biol..

[41] Pepys MB (1994). Human serum amyloid P component is an invariant constituent of amyloid deposits and has a uniquely homogeneous glycostructure. Proc. Natl. Acad. Sci. USA.

[42] Liberta F (2019). Morphological and primary structural consistency of fibrils from different AA patients (common variant). Amyloid.

[43] Schmidt M (2019). Cryo-EM structure of a transthyretin-derived amyloid fibril from a patient with hereditary ATTR amyloidosis. Nat. Commun..

[44] Scheres SH, Zhang W, Falcon B, Goedert M (2020). Cryo-EM structures of tau filaments. Curr. Opin. Struct. Biol..

[45] Bergström J (2005). Amyloid deposits in transthyretin derived amyloidosis: cleaved transthyretin is associated with distinct amyloid morphology. J. Pathol..

[46] Westermark GT, Fändrich M, Westermark P (2015). AA amyloidosis: pathogenesis and targeted therapy. Annu. Rev. Pathol..

[47] Kollmer M (2019). Cryo-EM structure and polymorphism of Aβ amyloid fibrils purified from Alzheimer’s brain tissue. Nat. Commun..

[48] Schweighauser M (2020). Structures of α-synuclein filaments from multiple system atrophy. Nature.

[49] Bansal A (2021). AA amyloid fibrils from diseased tissue are structurally different from in vitro formed SAA fibrils. Nat. Commun..

[50] Fändrich M, Schmidt M (2021). Methods to study the structure of misfolded protein states in systemic amyloidosis. Biochemical Soc. Trans..

[51] Read, C., Walther, P. & von Einem, J. Quantitative Electron Microscopy to Study HCMV Morphogenesis, in: *Human Cytomegaloviruses: Methods and Protocols, Methods in Molecular Biology* (Ed. Yurochko, A. D.) 265–289 (Springer US, 2021). 10.1007/978-1-0716-1111-1_14 33555592

[52] Read, C., Schauflinger, M., Nikolaenko, D., Walther, P. & von Einem, J. Regulation of human cytomegalovirus secondary envelopment by a C-terminal tetra-lysine motif in pUL71. *J. Virol*. **93**, e02244-18 (2019). 10.1128/JVI.02244-18 PMC6580969 30996102

[53] Kremer JR, Mastronarde DN, McIntosh JR (1996). Computer visualization of three-dimensional image data using IMOD. J. Struct. Biol..

[54] Kollmer M (2016). Electron tomography reveals the fibril structure and lipid interactions in amyloid deposits. Proc. Natl. Acad. Sci. USA.

[55] Feuz L, Leermakers FAM, Textor M, Borisov O (2005). Bending rigidity and induced persistence length of molecular bottle brushes: a self-consistent-field theory. Macromolecules.

[56] Annamalai K (2016). Polymorphism of amyloid fibrils in vivo. Angew. Chem. Int. Ed..

[57] Schindelin J (2012). Fiji: an open-source platform for biological-image analysis. Nat. Methods.

[58] Zheng SQ (2017). MotionCor2: anisotropic correction of beam-induced motion for improved cryo-electron microscopy. Nat. Methods.

[59] Zhang K (2016). Gctf: Real-time CTF determination and correction. J. Struct. Biol..

[60] He S, Scheres SHW (2017). Helical reconstruction in RELION. J. Struct. Biol..

[61] Emsley P, Lohkamp B, Scott WG, Cowtan K (2010). Features and development of coot. Acta Crystallogr. D. Biol. Crystallogr..

[62] Afonine PV (2018). Real-space refinement in PHENIX for cryo-EM and crystallography. Acta Crystallogr. D. Struct. Biol..

[63] Afonine PV (2018). New tools for the analysis and validation of cryo-EM maps and atomic models. Acta Crystallogr. D. Struct. Biol..

[64] Williams CJ (2018). MolProbity: more and better reference data for improved all atom structure validation. Protein Sci..

[65] Wu Z (2020). MASH explorer: a universal software environment for top-down proteomics. J. Proteome Res..

[66] Niedermeyer TH, Strohalm M (2012). mMass as a software tool for the annotation of cyclic peptide tandem mass spectra. PloS ONE.

[67] Gill SC, Von Hippel PH (1989). Calculation of protein extinction coefficients from amino acid sequence data. Anal. Biochem..

[68] Kabat. E. A., Wu, T. T., Reid-Miller, M., Peny, H. & Gottesman, K. *Sequences of Proteins of Immunological Interest*. 4th edn (United States Department of Health and Human Services, 1987)

[69] Fernandez-Escamilla AM, Rousseau F, Schymkowitz J, Serrano L (2004). Prediction of sequence-dependent and mutational effects on the aggregation of peptides and proteins. Nat. Biotechnol..

[70] Maurer-Stroh S (2010). Exploring the sequence determinants of amyloid structure using position-specific scoring matrices. Nat. Methods.

[71] Garbuzynskiy SO, Lobanov MY, Galzitskaya OV (2010). FoldAmyloid: a method of prediction of amyloidogenic regions from protein sequence. Bioinformatics.

[72] Conchillo-Solé O (2007). AGGRESCAN: a server for the prediction and evaluation of “hot spots” of aggregation in polypeptides. BMC Bioinform..

[73] Walsh I, Seno F, Tosatto SC, Trovato A (2014). PASTA 2.0: an improved server for protein aggregation prediction. Nucl. Ac. Res..

[74] Pettersen EF (2004). UCSF Chimera—a visualization system for exploratory research and analysis. J. Comput. Chem..

