# Supplementary material for: Role of mutations and post-translational modifications in systemic AL amyloidosis studied by cryo-EM
Source: Nat Commun. 2021 Nov 5;12:6434. doi: 10.1038/s41467-021-26553-9 (PMC8571268; doi:10.1038/s41467-021-26553-9)
Supplement: Supplementary file 1 — Supplementary Information [file 41467_2021_26553_MOESM1_ESM.pdf]

Supplementary Information

Role of mutations and post-translational modifications

in systemic AL amyloidosis studied by cryo-EM

Radamaker et al.

Supplementary Figures

Supplementary Figure 1

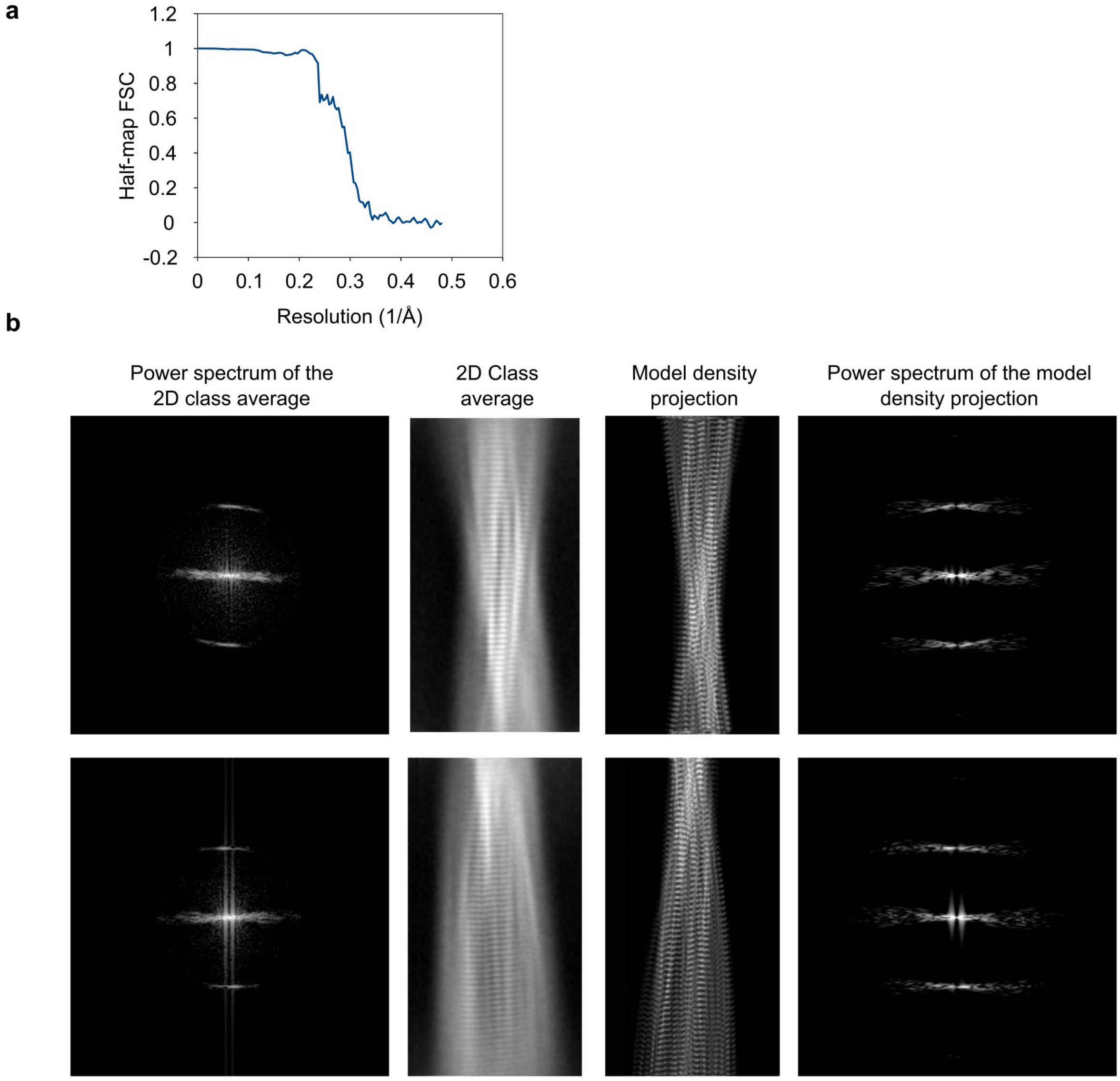

Supplementary Figure 1

FSC and comparison of the molecular model with the 2D class averages.

(a) Half-map FSC. (b) 2D classes (center left) compared with corresponding model projections (center right). The images at the far left and far right column show the power spectra of the 2D class

averages and model density projection as indicated. The top and the bottom rows refer to different z-axial positions in the fibril helix.

Supplementary Figure 2

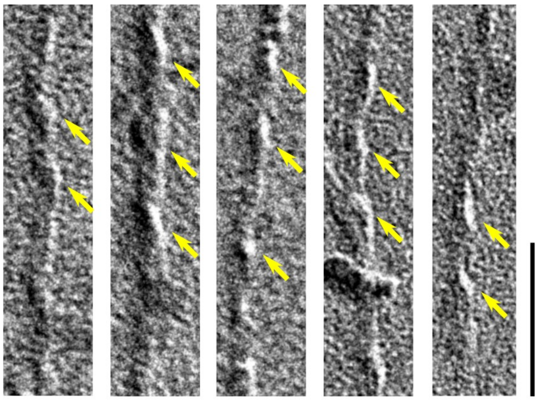

Supplementary Figure 2

Handedness of the FOR001 amyloid fibrils.

TEM images of a platinum side shadowed grid of FOR001 fibrils show a left-hand twist. To guide the eye, yellow arrows indicate the fibril crossovers. Scale bar is 100 nm.

Supplementary Figure 3

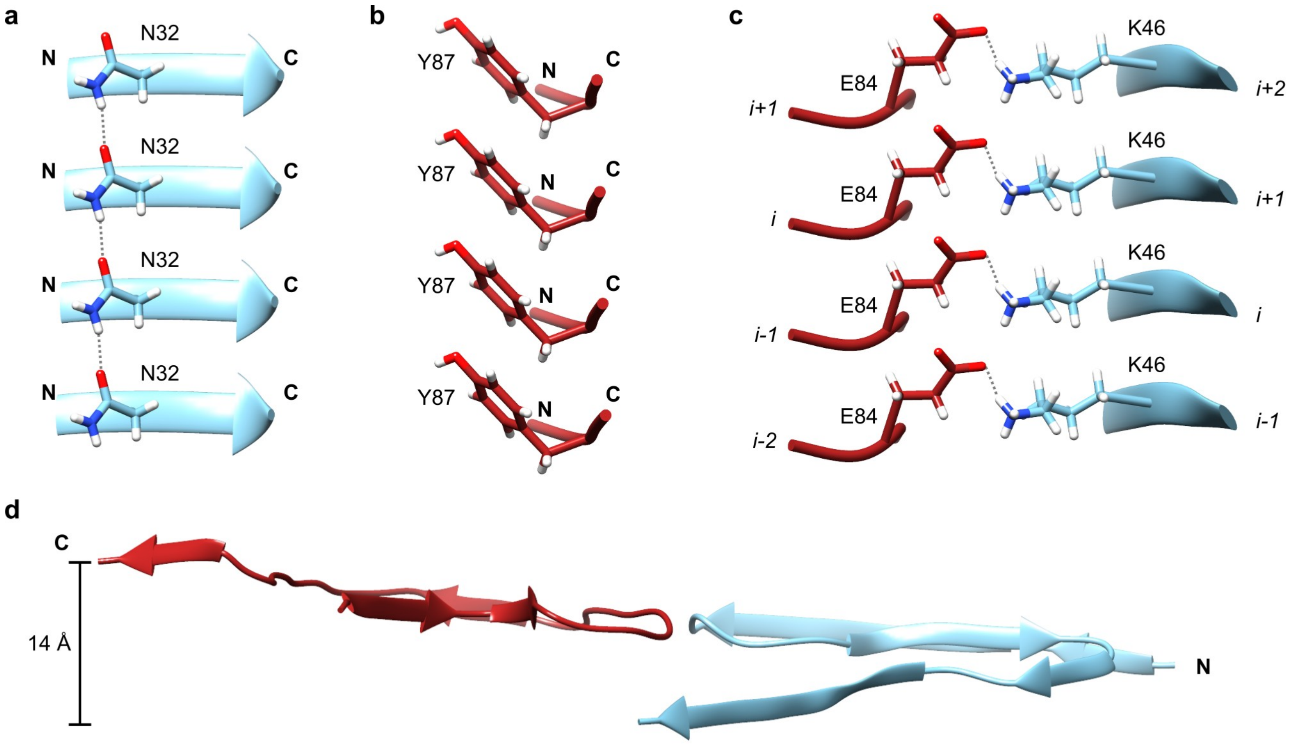

Supplementary Figure 3

Detailed views of the fibril structural model.

(a) Polar ladder formed by Asn32 residues. Dotted lines represent hydrogen bonds. (b) Aromatic stacking of Tyr87 residues. (c) Electrostatic interactions between Glu84 of layer *i* and Lys46 of layer *i+1*. Dotted lines represent salt bridges. (d) Side view of a single protein layer showing the height change of the protein backbone. The color code of the fibril protein in this figure corresponds to Fig. 1; that is, light blue refers to the N-terminal segment of the ordered fibril protein (residues Ser9-Thr52), while the deep red segment refers to the C-terminal segment (residues Ser68-Thr108).

Supplementary Figure 4

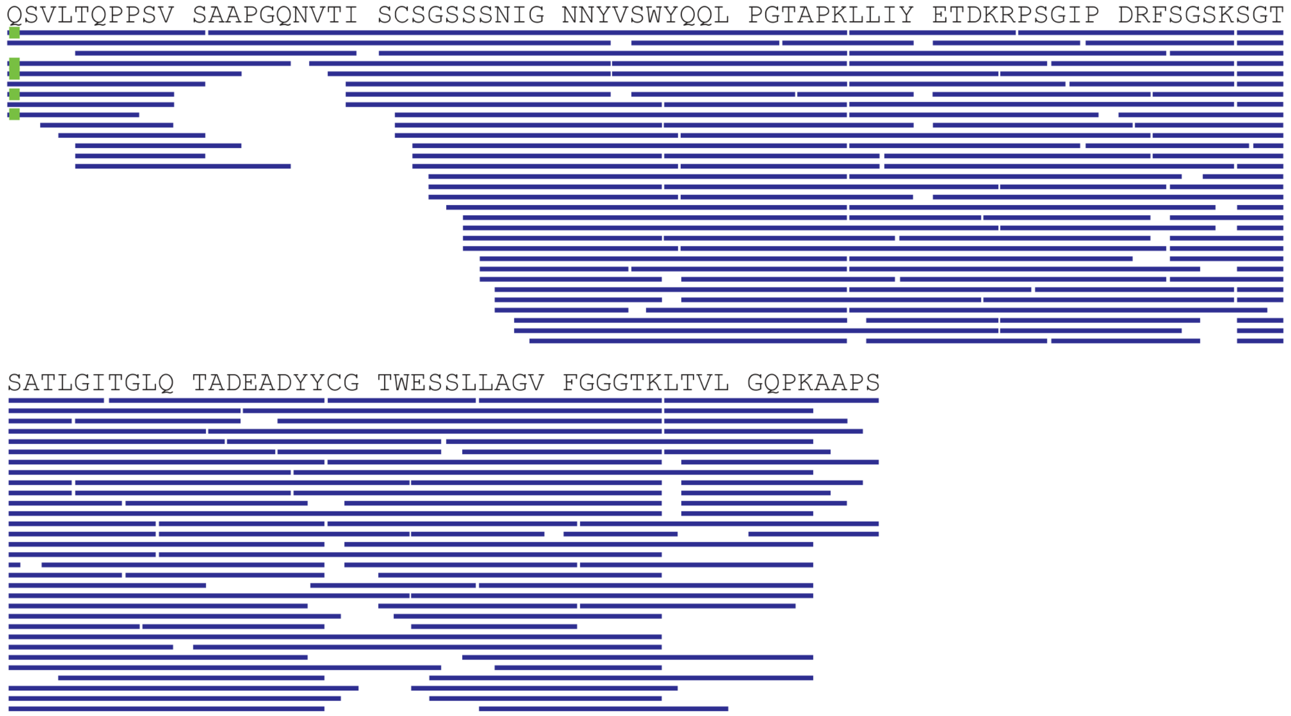

Supplementary Figure 4

Protein sequence of the FOR001 fibril protein.

Blue bars: peptide fragments identified by electrospray ionization MS and Peaks X software of glycosylated FOR001 fibril protein that was alkylated and treated with different proteases (see Methods for details). Green square: Pyro-Glu. Only a fraction of the identified peptides is displayed in the figure. Cys residues were uniformly found to be alkyated.

Supplementary Figure 5

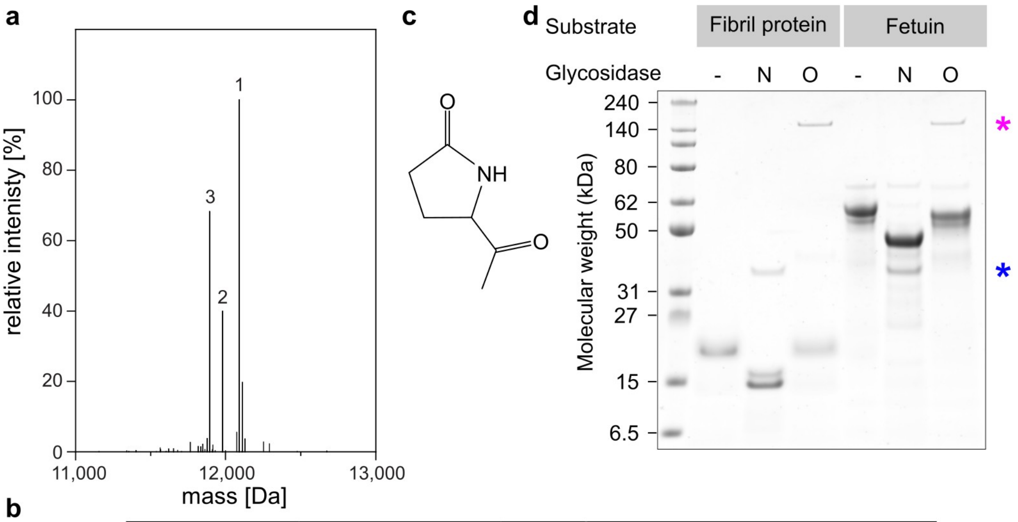

b

peak	experimental mass	theoretical mass	start	end	posttranslational modifications
1	12,089.9 Da	12,089.9 Da	Q1	S118	1x disulfide bond<br>1x pyroglutamylation
2	11,978.9 Da	11,978.9 Da	S2	S118	1x disulfide bond
3	11,891.9 Da	11,891.9 Da	S2<br>V3<br>L4<br>Q6	P117<br>S118<br>V119<br>L121	1x disulfide bond<br>———"———<br>———"———<br>———"———

Supplementary Figure 5

Mass of the deglycosylated FOR001 fibril protein.

(a) Deconvoluted mass spectrum of the deglycosylated FOR001 fibril protein. (b) Assignment of peaks 1-3 of the deconvoluted mass spectrum shown in (a). The removal of the glycosylation at position 17 converted Asn into Asp. Blue: assignment of fibril protein fragment. For peak 3, alternative assignments are possible (black). (c) Chemical structure of Pyro-Glu. (d) Denaturing electrophoresis gel showing the deglycosylation samples of the FOR001 fibril protein or fetuin incubated with N-glycosidase or O-glycosidase. The first lane is a size marker. Blue star: position of the N-glycosidase (PNGase F); magenta star: position of the O-glycosidase. Fetuin was used as a control substrate to demonstrate the activity of both enzymes. This gel was replicated at least three times.

Supplementary Figure 6

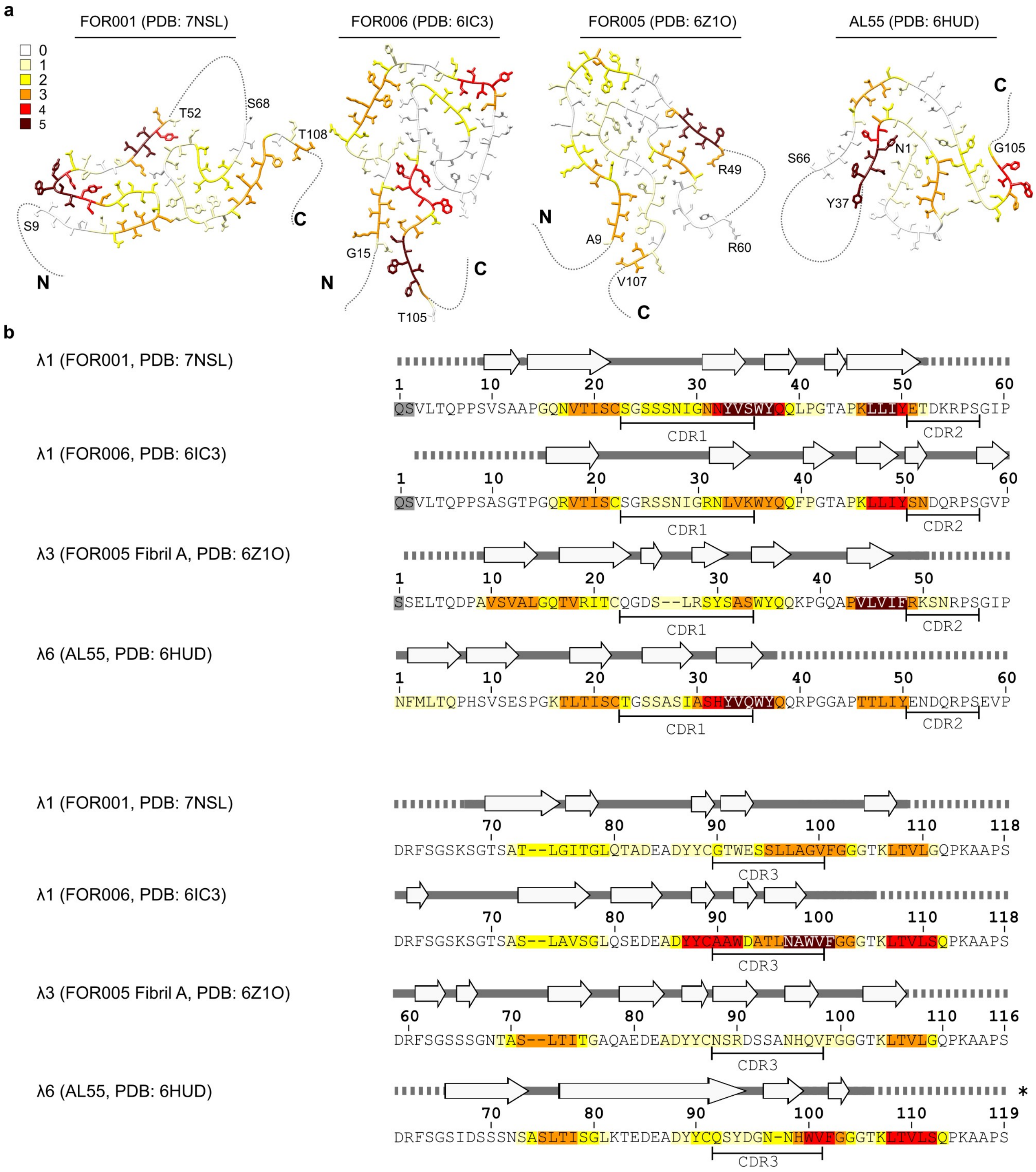

Supplementary Figure 6

Location of the aggregation prone segments in the available ex vivo fibril structures.

(a) Four published ex vivo AL amyloid fibril structures[1,2,3] colored according to aggregation score (0-5, as indicated in the figure). (b) Sequence alignment of the four fibril proteins[1,2,3]. The secondary structure of the fibril protein is displayed over the sequence. Continuous gray bar indicates the ordered segments of the fibril protein. Dotted lines indicate disordered segments that were not resolved by cryo-EM. White arrows refer to the location of the β-strands, as defined by the authors of each study. Star: no unique C-terminal end of the fibril protein reported. The sequences are color-coded according to the aggregation score as in (a). Gray boxes indicate N-terminal, cleaved residues.

Supplementary Figure 7

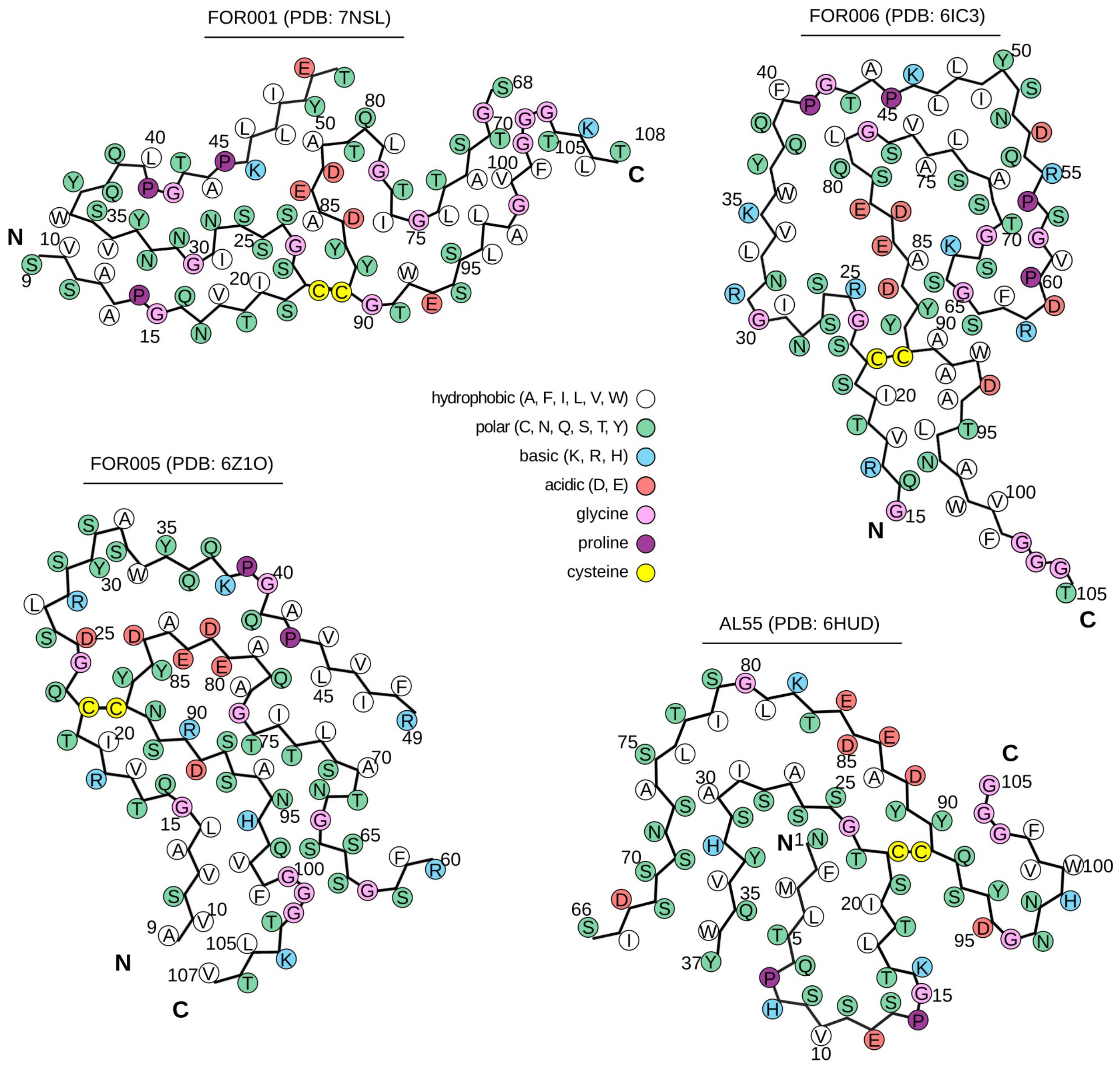

Supplementary Figure 7

Schematic comparison of four AL amyloid fibril folds.

Schematic representation of the FOR001 (PDB: 7NSL) fibril as compared to the previously reconstructed fibrils from patients FOR006 (PDB: 6IC3 [1]) and FOR005 (PDB: 6Z1O [2]) as well as of the AL55 fibril (PDB: 6HUD [3]).

Supplementary Tables

Supplementary Table 1

	(EMD-12570)<br>(PDB 7NSL)
**Data collection and processing**	
Magnification	130,000
Voltage (kV)	300
Electron exposure (e– $Å^{-2}$) per frame	1
Defocus range (μm)	0.41 – 8.6
Pixel size (Å)	1.04
Symmetry imposed	C1
Initial particle images (no.)	43,308
Final particle images (no.)	43,308
Map resolution (Å)<br>FSC threshold (0.143)	3.1
Rise (Å)	4.76
Twist (°)	-1.46
Pitch (nm)	117
**Refinement**	
Initial model used (PDB code)	De novo
Model resolution (Å)<br>FSC threshold 0.143<br>FSC threshold 0.5	 3.0<br>3.2
Map CC (mask)	0.77
EMRinger ZScore	6.79
EMRinger Score	3.83
Model composition<br>Non-hydrogen atoms<br>Protein residues<br>Ligands	 4263<br>595<br>0
B factors ($Å^2$)<br>Protein<br>Ligand	 53.74<br>-
R.m.s. deviations<br>Bond lengths (Å)<br>Bond angles (°)	 0.009<br>1.516
Validation<br>MolProbity score<br>Clashscore<br>Poor rotamers (%)	 1.13<br>1.44<br>0
Ramachandran plot<br>Favored (%)<br>Allowed (%)<br>Disallowed (%)	 96.30<br>3.70<br>0
B-factor after autosharpening ($Å^2$)	40.8

Contour level	12

Supplementary Table 1.

Reconstruction and modeling statistics.

Supplementary Table 2

	**FOR001 (this study)**	**FOR006 [1]**	**FOR005 [2]**	**AL55 [3]**
V segment	*IGLV1-51*02*	*IGLV1-44*01*	*IGLV3-19*01*	*IGLV6-57*02*
J segment	*IGLJ2 /IGLJ3*	*IGLJ3*	*IGLJ2*	*IGLJ3*
Number of residues in the V_L domain	110	110	108	111
Residues in fibril core	85	91	A: 89<br>B: 81	77
Residues in disordered V_L domain segments	25	19	A: 19<br>B: 27	34
Percentage of residues in ordered segments	77 %	83 %	A: 82 %,<br>B: 75 %	69 %
Number of mutations in the V and J segments	6	9	A: 6<br>B: 6	10
Number of mutations in ordered segments	5	9	A: 5<br>B: 5	5
Percentage of mutations in ordered segments	83 %	100 %	A: 83 %<br>B: 83 %	50 %

Supplementary Table 2

Comparison of the distribution of mutations and disordered residues in known fibril proteins. All analyses refer to residues within the V_L domain and hence to all residues of the full-length LC except from the signal sequence and C segment. Letters A and B in the FOR005 fibril refer to fibril conformations A and B [2]. The mean value and standard deviation of the percentage of disordered residues are 22 ± 6.2 % for the four fibrils. The mean value and standard deviation of the percentage of mutations in disordered segments are 21 ± 21 % for the four fibrils. The mean values are based on FOR005 structure A [2].

Supplementary References

[1] Radamaker, L., *et al.* Cryo-EM structure of a light chain-derived amyloid fibril from a patient with systemic AL amyloidosis. *Nat Commun* **10,** 1103 (2019)

[2] Radamaker, L., *et al.* Cryo-EM reveals structural breaks in a patient-derived amyloid fibril from systemic AL amyloidosis. *Nat Commun* **12,** 875 (2021)

[3] Swuec, P., *et al.* Cryo-EM structure of cardiac amyloid fibrils from an immunoglobulin light chain AL amyloidosis patient. *Nat Commun* **10,** 1269 (2019)
