# Supplementary material for: Role of mutations and post-translational modifications in systemic AL amyloidosis studied by cryo-EM
Source: Nat Commun. 2021 Nov 5;12:6434. doi: 10.1038/s41467-021-26553-9 (PMC8571268; doi:10.1038/s41467-021-26553-9)
Supplement: Supplementary file 2 — Reporting Summary [file 41467_2021_26553_MOESM2_ESM.pdf]

nature portfolio

Corresponding author(s): Marcus Fändrich

Last updated by author(s): 2021-09-20

Reporting Summary

Nature Portfolio wishes to improve the reproducibility of the work that we publish. This form provides structure for consistency and transparency in reporting. For further information on Nature Portfolio policies, see our Editorial Policies and the Editorial Policy Checklist.

Please do not complete any field with "not applicable" or n/a. Refer to the help text for what text to use if an item is not relevant to your study.
For final submission: please carefully check your responses for accuracy; you will not be able to make changes later.

Statistics

For all statistical analyses, confirm that the following items are present in the figure legend, table legend, main text, or Methods section.

n/a	Confirmed	
☐	☒	The exact sample size (*n*) for each experimental group/condition, given as a discrete number and unit of measurement
☐	☒	A statement on whether measurements were taken from distinct samples or whether the same sample was measured repeatedly
☐	☒	The statistical test(s) used AND whether they are one- or two-sided<br>*Only common tests should be described solely by name; describe more complex techniques in the Methods section.*
☒	☐	A description of all covariates tested
☒	☐	A description of any assumptions or corrections, such as tests of normality and adjustment for multiple comparisons
☐	☒	A full description of the statistical parameters including central tendency (e.g. means) or other basic estimates (e.g. regression coefficient) AND variation (e.g. standard deviation) or associated estimates of uncertainty (e.g. confidence intervals)
☐	☒	For null hypothesis testing, the test statistic (e.g. *F*, *t*, *r*) with confidence intervals, effect sizes, degrees of freedom and *P* value noted<br>*Give P values as exact values whenever suitable.*
☒	☐	For Bayesian analysis, information on the choice of priors and Markov chain Monte Carlo settings
☒	☐	For hierarchical and complex designs, identification of the appropriate level for tests and full reporting of outcomes
☐	☒	Estimates of effect sizes (e.g. Cohen's *d*, Pearson's *r*), indicating how they were calculated

Our web collection on statistics for biologists contains articles on many of the points above.

Software and code

Policy information about availability of computer code

Data collection	SerialEM v3.7
Data analysis	Relion v3.0, MotionCor2, Gctf v1.06, Coot v0.8.9, PHENIX v1.16, Molprobity, IMOD 4.9.0, Fiji (ImageJ) 1.52, UCSF Chimera v1.14, XCalibur 2.2 SP1.48, Peaks AB Software, Peaks X software suite, mMass, MATLAB 2019, MASH Explorer, TANGO v2.1, WALTZ, FoldAmyloid, Aggrescan, PASTA 2.0, abYsis

For manuscripts utilizing custom algorithms or software that are central to the research but not yet described in published literature, software must be made available to editors and reviewers. We strongly encourage code deposition in a community repository (e.g. GitHub). See the Nature Portfolio guidelines for submitting code & software for further information.

Data

Policy information about availability of data

All manuscripts must include a data availability statement. This statement should provide the following information, where applicable:
- Accession codes, unique identifiers, or web links for publicly available datasets
- A description of any restrictions on data availability
- For clinical datasets or third party data, please ensure that the statement adheres to our policy

The datasets used during the current study are available from public repositories and/or from the corresponding author on reasonable request. The cryo-EM map of the FOR001 fibrils was deposited in the Electron Microscopy Data Bank (https://www.ebi.ac.uk/pdbe/emdb/) with the accession code EMD-12570. The coordinates of the corresponding atomic model were deposited in the PDB (https://www.rcsb.org/) under the accession code 7NSL. The cryo-EM data of the FOR001 fibrils were deposited on EMPIAR (https://www.ebi.ac.uk/pdbe/emdb/empiar/) with the accession code EMPIAR-10730. The following published PDB structures were used in the manuscript: 4ODH 10.2210/pdb4ODH/pdb, 6IC3 10.2210/pdb6IC3/pdb, 6Z1O 10.2210/pdb6Z1O/pdb, 6HUD 10.2210/pdb6HUD/pdb, 5JZ7 10.2210/pdb5JZ7/pdb, 6QB6 10.2210/pdb6QB6/pdb, 6Q0E 10.2210/pdb6Q0E/pdb, 7JVA 10.2210/pdb7JVA/pdb, 5MUD 10.2210/pdb5MUD/pdb. The accession codes for the IGLV1-51*02 segment from the IMGT database is M30446 http://www.imgt.org/ligmdb/view?id=M30446 and from the VBase2 humIGLV015 http://www.vbase2.org/vgene.php?id=humIGLV015. The IGLJ2 and IGLJ3 gene segments were taken from GenBank with the Gene IDs 28832 https://www.ncbi.nlm.nih.gov/gene/28832 and 28831 https://www.ncbi.nlm.nih.gov/gene/28831, respectively. Source data are provided with this paper for the following figures: Fig. 1c, Fig. 5c,d,e, Supplementary Figure 1a, Supplementary Figure 2, Supplementary Figure 5d and Supplementary Figure 6.

Field-specific reporting

Please select the one below that is the best fit for your research. If you are not sure, read the appropriate sections before making your selection.

☒ Life sciences ☐ Behavioural & social sciences ☐ Ecological, evolutionary & environmental sciences

Life sciences study design

All studies must disclose on these points even when the disclosure is negative.

Sample size	Fibrils were extracted from a heart tissue sample of a single patient. 3,033 micrographs were collected from a single grid.
Data exclusions	Initially 279,338 segments were selected. In a more selective manual picking round aiming to select only fibrils from a single morphology based on width and crossover distance 43,308 segments were picked. All of these were used for the final reconstruction.
Replication	The images of 43,308 fibril segments are replicate measurements of the same fibril structure. The reconstruction is based on these replicates, therefore the reconstruction itself was not replicated. ThT measurements were replicated 3 times as indicated in Figure 5c. Each gel in the study was replicated at least once.
Randomization	Single case study of a patient-specific disease, therefore the results are not generalizable to other patient cases
Blinding	Experiments were performed on explanted heart tissue, therefore blinding was not relevant

Reporting for specific materials, systems and methods

We require information from authors about some types of materials, experimental systems and methods used in many studies. Here, indicate whether each material, system or method listed is relevant to your study. If you are not sure if a list item applies to your research, read the appropriate section before selecting a response.

Materials & experimental systems

n/a	Involved in the study	
☒	☐	Antibodies
☒	☐	Eukaryotic cell lines
☒	☐	Palaeontology and archaeology
☒	☐	Animals and other organisms
☐	☒	Human research participants
☒	☐	Clinical data
☒	☐	Dual use research of concern

Methods

n/a	Involved in the study	
☒	☐	ChIP-seq
☒	☐	Flow cytometry
☒	☐	MRI-based neuroimaging

Human research participants

Policy information about studies involving human research participants

Population characteristics	See Methods: Source of AL amyloid fibrils: Gender; Male; Age: 51 years; Diagnosis: AL amyloidosis
Recruitment	Selected based on clinical findings. Heart tissue was collected from a male patient (FOR001) at the age of 51, suffering from AL amyloidosis and consequent advanced heart failure. A monoclonal gammopathy was the underlying condition. The patient was treated within the heart transplant program of the University Hospital Heidelberg. This is a single case study
Ethics oversight	The study was approved by the ethical committees of the University of Heidelberg (123/2006) and of Ulm University (203/18). Informed consent was obtained from the patient for the analysis of the amyloid deposits.

Note that full information on the approval of the study protocol must also be provided in the manuscript.
